# Bioactive Compound-Loaded Nanocarriers for Hair Growth Promotion: Current Status and Future Perspectives

**DOI:** 10.3390/plants12213739

**Published:** 2023-10-31

**Authors:** Arvind Sharma, Harapriya Mohapatra, Kanika Arora, Ritchu Babbar, Rashmi Arora, Poonam Arora, Pradeep Kumar, Evren Algın Yapar, Kailash Rani, Maninder Meenu, Marianesan Arockia Babu, Maninderjit Kaur, Rakesh K. Sindhu

**Affiliations:** 1 School of Pharmaceutical and Health Sciences, Bhoranj (Tikker–Kharwarian), Hamirpur 176041, India; arvind.pharmacy@gmail.com; 2 Chitkara College of Pharmacy, Chitkara University, Rajpura 140401, India; priya20003.ccp@chitkara.edu.in (H.M.); kanika20004.ccp@chitkara.edu.in (K.A.); ritchu.babbar@chitkara.edu.in (R.B.); rashmi.arora@chitkara.edu.in (R.A.); poonam.arora@chitkara.edu.in (P.A.); vkailash15@gmail.com (K.R.); 3 Wits Advanced Drug Delivery Platform Research Unit, Department of Pharmacy and Pharmacology, School of Therapeutic Sciences, Faculty of Health Sciences, 7 York Road, Parktown, Johannesburg 2193, South Africa; pradeep.kumar@wits.ac.za; 4 Faculty of Pharmacy, Sivas Cumhuriyet University, Sivas 58140, Türkiye; evrenalginyapar@cumhuriyet.edu.tr; 5 Department of Agri-Biotechnology, National Agri-Food Biotechnology Institute, Mohali 143005, India; meenu_maninder@yahoo.com; 6 Institute of Pharmaceutical Research, GLA University, Mathura 281406, India; babuphd2001@yahoo.co.in; 7 Department of Pharmaceutical Sciences, Lovely Professional University, Phagwara 144411, India; maninder13chd@gmail.com; 8 School of Pharmacy, Sharda University, Greater Noida 201306, India

**Keywords:** hair growth, phytoconstituents, nanocarriers, nanoarchitectonics technology, molecular mechanisms

## Abstract

Hair loss (alopecia) has a multitude of causes, and the problem is still poorly defined. For curing alopecia, therapies are available in both natural and synthetic forms; however, natural remedies are gaining popularity due to the multiple effects of complex phytoconstituents on the scalp with fewer side effects. Evidence-based hair growth promotion by some plants has been reported for both traditional and advanced treatment approaches. Nanoarchitectonics may have the ability to evolve in the field of hair- and scalp-altering products and treatments, giving new qualities to hair that can be an effective protective layer or a technique to recover lost hair. This review will provide insights into several plant and herbal formulations that have been reported for the prevention of hair loss and stimulation of new hair growth. This review also focuses on the molecular mechanisms of hair growth/loss, several isolated phytoconstituents with hair growth-promoting properties, patents, in vivo evaluation of hair growth-promoting activity, and recent nanoarchitectonic technologies that have been explored for hair growth.

## 1. Introduction

Approximately 30–50 percent of males by the age of fifty and 12–40 percent of women are affected by androgenetic alopecia (AGA), also referred to as male and female pattern alopecia, respectively. The androgen impact on genetically susceptible follicles’ epithelium in androgen-dependent zones causes gradual shrinkage of the follicles [1,2,3,4].

While aging and complicated genetic inheritance are key risk factors for AGA progression, the commencement of an inflammation-causing stage in the follicular microenvironment is the principal locus of occurrence, involving processes such as aberrant transcriptional activation among the contributors [5]. Oxidative stress and apoptosis are both amplified. Hair loss is caused by several different mechanisms, including 5-dihydrotestosterone (DHT), inflammation, and oxidative stress, all of which contribute to the loss of keratinocytes. A multifaceted strategy for treating AGA is required to resolve both issues. Dermal papilla cells (DPCs) produce interleukin (IL-6) and converting growth factor (TGF-2) in response to DHT, which inhibits hair development and causes the catagen stage to begin prematurely in AGA patients [6,7,8,9,10,11].

At this stage, AGA is not cured. The most prevalent side effects among people with AGA include anxiety and depression. Orally administered finasteride and topical minoxidil have already been approved for AGA in the USA. However, finasteride was confined to men only, as teratogenicity in pregnancy and poor post-menopausal effects were observed in women [12,13,14]. Whilst the molecular mechanisms of minoxidil are undetermined, it is expected to reduce the telogen period and enhance the telogen–exogen period [15]. When testosterone is converted to its activated state (DHT), the end step in the process, finasteride can be used as a type II 5-alfa-reductase inhibitor [16]. Baldness can be slowed, and new hair regrowth can be accelerated with the use of these two drugs. However, they perform at a below-average level [17,18]. It has been found that only 48.06% and 38.66% of the participants who took oral finasteride for one year and four months showed hair extension [19]. Additionally, a research study found that finasteride had no effect on hair loss in postmenopausal women. Topical minoxidil has already been linked to incidences of scalp irritation and inadequate efficacy in some individuals [20,21]. Due to these many side effects, there is a great demand for plant substitutes for the management of alopecia. Hence, there is an unmet demand for additional safe and reliable AGA treatments.

There are various causes of baldness that can result from constant and persistent pressure on the hair, including traction alopecia (TA). Hairstyles that pull significantly at the frontotemporal horizon are the most frequent source among children and women of African descent [22]. The frontal and temporal areas, as well as the areas above and below the ears, are frequently affected by TA. In addition to the hairstyles, TA patients have also reported wearing their hair in tight weave or braid configurations, or in ponytails. Consider the possibility of TA when applying traction to hair that has been treated with relaxers or colors [23]. The clinical diagnosis of TA may be aided by the presence of retained hairs around the frontal and/or temporal hairlines (the “fringe sign”) [24,25,26,27]. Corticosteroids focused on the periphery of hair loss, antibiotics with anti-inflammatory drugs shortly after TA onset, minoxidil, and hair implantation have all been utilized as treatments for TA, as well as suppressing all events that cause the condition.

In terms of the longevity of baldness, central centrifugal cicatricial alopecia (CCCA) is second to TA in order of significance in the occurrence of alopecia in women of African descent, with a recurrence of 3–7 percent, although the exact incidence in varied groups is unknown [28]. Scientists have linked CCCA to a mutation in the PAD13 gene, which encodes proteins essential for the proper development of the hair shaft. A genetic predisposition to CCCA can be triggered using abrasive hair grooming procedures [29]. In most cases, CCCA-induced scarring alopecia develops on the vertex of the scalp in a centrifugal configuration with symmetrical spread. Dermoscopy indicates the presence of a white halo at the periphery of the eye, as well as other findings. CCCA is assumed in an African American patient with thinning of the middle portion of the body. Treatments like those used for TA, as well as doxycycline and hydroxychloroquine, have been employed, but many are ineffective and unsatisfactory [30].

There are many different types of botanical extracts that may be made from a variety of different plant organs such as flowers or fruits. They can be applied topically or ingested. These extracts contain a wide range of phytochemicals and unknown pollutants. Their medicinal use has increased dramatically in the past few decades. Due to its wide accessibility and inexpensive cost, this approach is promising because it can be used to create many different products that can target certain disease-related abnormalities. Nevertheless, some phytochemicals are subject to laws or intensive research because they are not licensed for a specific ailment [31]. Consequently, their efficacy and safety are hotly contested.

## 2. Human Hair Development in the Embryo—Molecular Mechanisms

At the ninth week of intrauterine life, the human hair follicle undergoes four unique phases of development. Interactions among epidermal placodes and dermal cells are critical for hair follicle production in various tissue types [32,33,34], including the nails, teeth, and most exocrine glands [35,36,37]. This communication is assumed to be mediated by a variety of signaling mechanisms. Wnt Hedgehog, TGF-/BMP, fibroblast growth factor (FGF), other tumor necrosis factors (TNFs), and paracrine signaling factors are believed to play critical roles in the development of cancer and most exocrine glands [36,37]. Anagen (proliferation), catagen (regression), telogen (relax), and exogen (exfoliating) are the four major stages of the hair follicle cycle. Anagen is a time when the hair is actively growing. In early anagen, the hair matrix produces new hair. A steady supply of blood nourishes hair follicles. Anagen has a life expectancy of two to six years. Catagen is the intermediate phase in which collapse of the hair follicle’s deepest section causes the hair follicles (HFs) to become disconnected from the feeding blood supply. It lasts between one and two weeks. In telogen, hair mass remains dormant; papillary cells have separated from HFs and are inactive during the five- to six-week resting phase. Exogen is the phase of shedding in the last stages of life when hairs shed. However, it can also occur in the telogen or anagen phases.

Throughout postnatal life, the hair follicles of mammalian skin undergo frequent intricacy and regeneration cycles [38]. Anatomical locations, dietary and hormonal conditions, age, and species all influence the tenure of each phase. [39]. Since the first test and hair shaft in mice are not formed until 17days after birth [20], it is often mistaken for the first anagen in human studies [40,41]. The follicles of the scalp go through 10–30 cycles throughout their lives. White adipose tissue, which forms dermal cones surrounding pilosebaceous units, is hypothesized to have a role in human HF cycling [42].

### 2.1. Anagen

There are four weeks of real anagen following birth, which is when the body begins to expand. Proliferating stem cells in the bulge area produce a new lower hair follicle at the anagen stage [43]. Human hair follicular bulging cells or units have keratin fifteen and integrin [44]. When produced from epithelial high-frequency stem cells in the bulge, the hair matrix temporarily multiplies cells similarly to proliferate and then differentiate into discrete epithelial hair lineages [45]. The hair follicle stem cells are, however, exceedingly slow at cycling throughout the anagen, catagen, and telogen development stages [46,47].

### 2.2. Catagen

Over the course of 2–3 weeks, the bottom two-thirds of the HF gradually degenerate, leaving just the club hair surrounded by an epithelial cap. Inner and outer root keratinocytes are undoubtedly essential. Stem cells from the HF bulge are spared [48]. Finally, an epithelial strand is formed, which is an epithelial HF remnant that serves to resemble the dermal papilla bulge [36]. The old hair layer becomes visible as novel hairs emerge from the same follicle. ORS may bulge around a mouse club hair if it rests in the slot for multiple cycles, which increases the coat density [49].

### 2.3. Telogen

**Figure 1:**
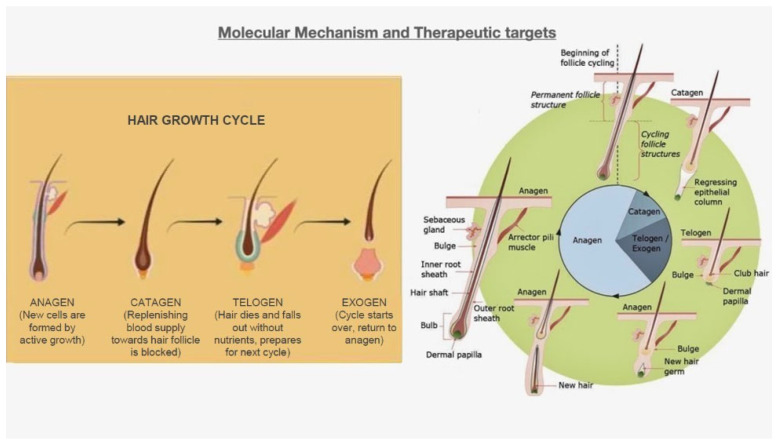
Molecular mechanisms and drug targets.

Telogen is the repose phase of the hair follicle cycle and is associated with exfoliation or baldness. It follows the catagen phase (Figure 1) [33]. At an early stage of development, the hair follicle cycling of the mouse epidermis is well synchronized, but this coordination diminishes with maturity [29]. The human hair follicle cycles on both sides begin to desynchronize soon after birth. In addition, the time spent in telogen lengthens with age [50,51], with a slower rate of hair follicle turnover in both humans and animals. Anagen/telogen ratios are responsible for the wide range of hair lengths seen on the human body (including eyelashes, the chest, and the scalp). Long hair grows on the scalp because of the large anagen/telogen ratio, while limb hair and eyelashes spend longer in telogen and less duration in anagen [52].

### 2.4. Exogen

Exogenous hair loss is generally an evolving process, but exogenous hair shedding may occur passively due to mechanical factors (Figure 1) [43]. At various times, human HFs go through the four cycles. There are approximately eighty-six percent of hairs in the anagen phase, one percent of hairs in the catagen phase, and just thirteen percent in the telogen phase at any one time in the human HF cycle [53]. The hair follicle stem cells are found in the ORS bulge [54]. Homeostasis slows bulk cell cycling and inactivity [45,46,47]. Different from stem cells in other tissues, these are biologically different and survive throughout life. Permanent and chronic alopecia may be caused by inflammation-induced bulge damage and bulge cell death [55]. Human bulging epithelial stem cells are hypothesized to be identifiable by the expression of keratin fibers K15 and K19, enhanced CD200 expression, and reduced expression of CD34, connexin, and nestin 43 [56,57]. Anagen, which produces daughter cells through asymmetric division, begins when the bulge stem cells begin to proliferate. The offspring cells of the stem cell penetrate the spontaneously energized matrix cells that can arise due to the adolescent hair follicle’s cell cultures when they reach the hair bottom [58,59]. Hair follicle stem cells have the potential to repair sebaceous glands as well as the interfollicular epidermis [60]. Stem cells in the HF population have been shown to generate melanocytes, brain stem cells, and keratinocytes throughout life [38].

## 3. Follicle Cycle Mechanisms in Hair

The molecular processes behind hair follicle cycling are still a mystery. Hair follicle development in humans is now being studied in more detail, which has laid the groundwork for understanding crucial regulations [61].

### 3.1. Anagen

Stem cell incoherence and persistence may be fine-tuned by gene transcription monitoring in both mice and humans [62]. Wnt, activin/BMP, and TGF-/BMP signaling in mouse and human bulging cells, as well as noggin, FGF, and sonic hedgehog (Shh) antagonists, play a significant role in anagen production [63]. Elevated Wnts, the stability of β-catenin, bone morphogenetics protein suppression by antagonists, and elevated levels of c-myc and Runx1 inside the hair follicle bulge cells contribute to the activation of stem cells [64,65,66,67]. Because of the abnormal proliferation of HF stem cells in mice and missingBMPR1a, these animals eventually lose their slow-cycling cells [68]. In these animals, the transcriptional lymphoid enhancer binding factor-1 (Lef-1) and stabilized β-catenin within the environment of stem cells are elevated and abnormal. DIO2 and ANGPTL2 expression have been shown to be upregulated only in humans [69,70]. Mice and humans differ in the accumulation of latent TGF-binding protein 2, CD34, and FGF18 [36]. Hepatocyte growth factor and vascular endothelial growth factor (VEGF) are responsible for maintaining anagen [71]. Bulge stem cells remain dormant as anagen develops because Wnt inhibition via TCF3, Wnt inhibitory factor 1 and Dkks (e.g., Dkk3), and the calcium-dependent transcription factor NFATc1 [72] restore the bulge to a Wnt-inhibited state [73]. As a result of BMP signaling, Wnt pathway activation is prevented, and the HF stem cells are kept in quiescence. Anagen maintenance is regulated by P-cadherin, which regulates canonical Wnt signaling and suppresses TGF-2 in organ-cultured human scalp hair follicles. The daughter bulge cells move away from the dermal papilla when the epithelial hair follicle stem cells return to quiescence [74]. The anagen follicle’s specialized cell fate is likewise determined by Wnt signaling [75]. Srfp1, Dab2, and TCF3 inhibitors have been shown to be diminished in non-bulge keratinocytes compared to bulging stem cells in human and mouse skin [76]. Wnt signaling is kept active and β-catenin is stabilized during anagen by the transient multiplying cells [77,78]. After a certain point, the precortical hair matrix cells stop growing and diverge into distinct terminal hair follicle epithelial cell lines [36,79]. When it comes to ORS creation, BMPs, GATA3, and Cutl1 are implicated, whereas IRS formation is facilitated by Shh and Sox9. Wnt/β-catenin, BMPs, the vitamin D receptor (VDR), notch, and Foxn1 regulate hair shaft growth and keratin synthesis. For all epithelial lineages, TCF3 acts as a generic inhibitor of TCF3 [80].

### 3.2. Catagen

TNF-signaling downstream effectors, including VDR, keratin fiber K17, the retinoic acid receptor, and the transcriptional repressor Hairless, all play a role in the anagen-to-catagen shift. Mice lacking Hairless or VDR have HFs that develop into epithelial vesicles and dermal cysts when they enter catagen [81], which disturbs the crucial connection between hair follicle stem cells and the induced hair follicle mesenchyme, which is necessary for the proper function of HFs. Catagen onset is accelerated by FGF-5, a powerful factor [33]. FGF-5-deficient animals have an extended anagen and an angora phenotype (an exceptionally broad body surface hair sheath) [82], supporting the involvement of FGF-5. Gene mutations in FGF5 have been linked to a dramatic increase in eyelash growth, a condition known as familial trichomegaly [83]. In addition to interleukin-1α, neurotrophins-3, -4, and -5, BMP2 and BMP4 [36], and TNF-α [36], other variables work together to enhance catagen onset. The dermal papillary cells of human hair follicle cells treated with all-trans retinoic acid undergo an overexpression of TGF-2, resulting in a catagen-like state [84].

### 3.3. Telogen

Research in mice has shown that telogen can be divided into two distinct phases: the refractory phase, in which hair follicles are impervious to growth stimuli and have increased BMP2/4, and the competent phase, in which hair follicle bulge stem cells are hypersensitive to anagen-inducing factors, BMP signaling is diminished, and Wnt/-catenin signaling is increased [85,86]. It is worth noting that the estrogen receptor is significantly upregulated during telogen. These two systems must work together to guarantee that individual hair follicle cycles are regulated, and this might be related to the finding that bone morphogenetics protein (BMP2) and morphogenetics protein (BMP4) expression goes through cyclical variations in the extrafollicular dermis, notably in subcutaneous adipocytes. The bulging stem and dermal papilla cells may communicate [87] and assist the activation of stem cells in concert for the commencement of a new hair follicle cycle, while the dermal papilla sits immediately below the bulging cells in telogen. 

## 4. Recent Nanotechnology-Based Formulations for Human Hair Growth

Hair regrowth starts anew after the stem cell density reaches a threshold level [88]. In epithelial HF stem cells, several genes have been found to influence their ability to divide and their ability to undergo HF cycling. Hair follicle cycle monitoring and mouse genetics research will proceed to shed light on the mechanisms of the hair cycle clock in the future [89]. Weak hair may be caused by a range of medical disorders, including hormone imbalances, age-related changes in hair texture and density, autoimmune diseases, drugs, and heredity [90]. Researchers believe that hair loss might be caused by weakened or ruined hair stem cells. Tissue may be depleted in scarring alopecia, but this is not the case with baldness, which is caused by problems with stem cell maintenance and the depletion of progenitor cells. Allogeneic hair transplantation and medicines have made significant progress in the treatment of alopecia, but most of these attempts have failed to produce enough hair to be helpful. DPCs and epithelium-derived follicular stem cells have been transplanted, which is consistent with the necessity for reciprocal interactions for hair follicle morphogenesis and hair cycle progression [91,92]. The functional epithelial stem cells are thought to receive essential signals from the DPCs to control their development and to eventually determine the form, size, and color of the hair follicles [37]. This potential to proliferate and regenerate finally runs out in culture [93]. Particles that have shown promising results thus far include FGF-2 [94], Wnt [95], and BMP. Adding specific growth factors may assist in boosting hair creation efficiency [96]. DPC-specific indicators may be maintained by aggregating DPCs, and 3D preparations of HF germ cells, including epithelial cells and mesenchymal cells, resulted in effective hair follicle regeneration in mice [97]. It is possible to generate a significant number of hair follicle germ cells using this strategy, but it is time-consuming. To generate adequate quantities of strong stem cells for hair-regeneration therapy, recent research has concentrated on enhancing the efficiency of procedures [98]. While hair follicle enlargement is like wound healing in that it needs close coordination between tissue repair, cell proliferation, and cell migration, it also differs significantly. Throughout wound healing, hypoxia-inducible factor-1a promotes neovascularization, collagen, and elastin synthesis [99]. Human hair follicle stem/progenitor cells are hypoxia-responsive, and activating the hypoxia inducible factor-1 signaling pathway dramatically enhances the regrowth of cells, tissues, and hair development [100,101,102,103,104,105]. On the other hand, minoxidil, a hair growth stimulant, suppresses the HIF-degrading enzyme prolyl hydroxylase, which has a positive impact on hair development via the amplification of the angiogenic hypoxia-inducible factor-1a–vascular endothelial growth factor axis when given topically to the scalp (Figure 1). Research into HIF-modulating drugs might lead to new hair growth-stimulating therapies according to these results. It has been shown in mice that after cutaneous injury, Shh levels rise, which activates the hedgehog pathway and restores the skin restorative dermal niche (the epidermis papilla), which is required for HF neogenesis. This suggests that activating Shh signals in Wnt-responsive cells enhances wound healing, whether expressed excessively in the epidermis or via inherent smoothened dermal activation. When it comes to promoting hair follicle development, TGF and nerve growth factor families have contradictory roles; both promote HF development, but they also induce catagen in adult hair follicles. VDR, Hairless, and notch do not play a role in inducing anagen production in newborn skin; however, they are essential for HF development [106]. Various formulations for hair growth detailed in Table 1 with their mechemism of action.

The biological source of *Nardostachys jatamansi* (vernacular name: Jatamansi Spikenard or Jatamansi) is *Nardostachys jatamansi*, and it belongs to the Valerianaceae family. Jatamansi is composed of Jonon, 1,8 cineol, and bornyl acetate. This herb has been clinically proven to promote hair development. Jatamansi in an ethanolic extract that has been shown to have a possible hair growth effect in alopecia caused by chemotherapy. 

The herbal component of *Cuscuta reflexa* (Roxb. Amar Bel, Giant Dodder) is a parasitic, perennial herb that is generally leafless and yellowish–golden in color. This is often utilized in herbal medicines to impart medicinal activity. Chemically, this plant contains the following phytoconstituents: coumarin, amarbelin, sitosterol, dulcitol, quercitin, kaempferol, and others. It has been claimed that this herb has beneficial qualities in androgen-induced baldness, primarily in petroleum ether extract. 

Shrubby sophora is a common name for Aiton, Sophora flavescens. This plant belongs to the Leguminosae family. This is essentially a prehistoric Chinese therapeutic treatment. These plants mostly include flavonoids as a chemical component. It has been stated that when administered as an extract in conjunction with several growth hormones, including KGF and IGF-1, this plant promotes hair development. These, along with dermal cells, have been discovered to aid in hair growth. 

Onion is a common name for the herb *Allium cepa* L. The onion bulb is of the genium genus and cepa species and belongs to the Amaryllidaceae family. This bulb is primarily high in protein, specifically albumin. Allin, allyl propyl disulfide, allicin, and allyl sulfides are also found as chemical components. In addition, numerous mineral elements, such as zinc (Zn), magnesium (Mg), potassium (K), and calcium, are present (Ca). This allium species has been discovered to be beneficial in the treatment of baldness. Along with the use of honey, the extract or juice is placed topically on the scalp until it turns red. 

*Eclipta alba*, False Hassak (vernacular name: Daisy Bhringraja) is also known as *Eclipta alba*. This is an annual and tiny herb with white flowers on top that belongs to the Asteraceae family. It has been shown in prehistoric times to stimulate hair growth and prevent hair loss. 

*Umbellatus polyporus* is a mushroom that grows on maple trees. It is made up of steroidal and polysaccharide components. Another investigation discovered regrowth components such polyporusterone a and b and acetosyringone. In vitro investigation have revealed a significant increase in hair growth at substantially lower dosages of 1.28 and 6.4 g/mL, but higher doses inhibit hair development. 

Thunb knowgrass and knotweed are common names for *Multiflorum polygonium* plants. This Chinese medication is primarily used as a hair tonic and has anti-wrinkle and anti-aging properties. Most of the time, root tubers are used. This natural component is primarily used to prevent premature hair loss and graying of the hair. 

Tridax Daisy/Coatbuttons are a common names for Linnaeus’ Tridax procumbens. In India, Tridax procumbens, also known as Ghamra, is used for its flowering tops. This botanical component is widely employed in the old Ayurvedic system for illness issues. Chemically, it is made up of fumaric acid, tannins, flavonoids, glucoluteolin, procumbenetin, and quercetin. This plant’s leaves can heal a variety of ailments, including dysentery, bronchitis, and diarrhea, as well as prevent hair loss. Fruits of *Emblica officinalis* are high in vitamin C, tannins, and minerals such as phosphorus, iron, and calcium, which nourish the hair while also coloring it. Different patented formulations related to hair growth are tabulated in Table 2 and various animal models for the evaluation of hair growth formulations are given in Table 3.

Multi-volume inorganic halloysite clay nanotubes (HNT) have an outer diameter of 50–70 nm, an interior diameter of 10–20 nm, and a length of 500–1000 nm [179,180,181,182]. These rolled aluminosilicate sheets, which are phyllosilicates, are long, thin cylinders. An increase in the distance between spiral sections from 0.7 nanometers up to 1 nm [183] is like several naturally occurring minerals. By chemically exfoliating alumina and expanding the lumen’s diameter, the lumen volume can be enhanced for greater dye and medication loading capacity [184]. In contrast to the organizational chemistry of SiO_2_ (pH 4–8.5), Al_2_O_3_ results in the tubes’ charges being in opposition to one another. Halloysite surfaces have a structure that provides them with a preference for charged molecules, boosting the loading of negative substances into the bottle’s lumens and positive components onto the outer surface [185].

Self-assembly of the kaolin nanotube for cosmetic purposes has been investigated in a recent report. Using this method, nanotubes self-assembled on the hair’s outer surface and were loaded selectively into the lumens, allowing for long-term release of active colors. Mesoporous cuticles that surround the hair’s surface are exploited in the procedure, which utilizes the hair’s meso-porous nature. Hair can be washed for three minutes with one wt. percentage of water halloysite dispersion, which coats the cuticle folds with clay. Halloysite dispersion enters the inter-cuticle areas through the cuticles, which expand like flower petals in an aqueous system. Micro-confinement aligns the pipes under the impact of capillary pressure during evaporation. A sorbent may be required to dissolve halloysite-encapsulated dyes. It is possible to load kaolin nanotubes from any solution, which can be potentially dangerous, but after the formulation is complete, safe aqueous dispersions are used to administer these colored nanopigments to the hairs. 

Infestations caused by human lice can be eliminated with anti-lice medications [186]. Human pediculuscapitis is extremely significant. Challenges with conventional anti-lice compositions include resistance to common pesticides such as pyrethroids and permethrin, as well as re-infestations [187]. Delivery of anti-lice drugs must be continuous and hair-targeted to achieve efficiency. Hair dyes made from graphene-based carbon compounds have also shown potential. Graphene oxide and reduced graphene oxide are sheets made from graphite particles exfoliated in the presence of oxidizing agents. Animal models for the evaluation of hair growth formulations are reported in (Table 3).

There are now color nano-formulations made from a graphene-based nanosheet mixture and chitosan. As a result of this study, a toxicity-free method for coloring light-colored hairs with dark colors of brown to black was demonstrated. Antistatic and thermal dissipation properties, as well as resilience to several shampoos, were all demonstrated by these compositions [188]. 

For the last two decades, nanotechnology-based cosmetic and healthcare formulations have gained prominence. Nanomaterials are employed as functional coatings and carriers to treat and protect the hair shaft from external damage and to enhance the entry of active substances via the follicular route [189]. Nanostructured materials for topical medication administration allow regulated release over a long period of time, enhancing retention and reducing side effects and irritations. Nanomaterials include protein-based [190], natural [191,192,193], and synthetic polymers [194,195]; lipid-based [196,197], metallic [198,199], and silica nanoparticles [200,201]; and dendrimer [202], clay [203], and carbon nanotubes [204]. When employing nanomaterials for hair alteration, it is important to evaluate each method’s safety and consider ethnic differences [205].

The development of new nanomaterials for hair care has centered on improving the effectiveness of hair cosmetics by providing an instant or long-term impact, improved interaction, and better targeting. Furthermore, encapsulating cosmetic ingredients in nanoparticles enables the delivery of insoluble substances. To achieve target distribution, better stability has been proposed for many bioactive carriers of nano-based cosmetics formulations such as liposome micro- and nano-emulsions, niosomes, etc. Silicone oil, which is extensively used in cosmetic preparations for lubrication, is utilized to give conditioning effects to shampoos [206]. Silicone oil is intended to target the hair shaft rather than the hair skin over time. Silicone oil in nanostructures diffuses into the hair shaft following washing without damaging the cuticle. Oil-in-water emulsification using nonionic surfactants (Span 80 and Tween 80) was employed to create thermodynamically stable nano emulsions. X-ray and SEM examination showed silicon from nano emulsions on the hair shaft [207]. Cationic nano-emulsions (droplet diameter less than 100 nm) considerably improved the texture of dry hair, making it glossy, non-greasy, and less brittle [208]. Some researchers have shown that cosmetic nano compositions of oxides, silicates, hydroxides, phosphates, and carbonates can prevent hair from looking greasy [209]. Epoxy silicone nano emulsions (with a mean particle size of 100–250 nm) generated through micro fluidization is used to repair damaged hair (e.g., hair treated with chemical procedures such as hair lightening/bleaching, relaxing, dying, permanent waving, and hair smoothing). Epoxy silicone nano-emulsions have been shown to provide better strength, flexibility, and fatigue resistance than an untreated control [210]. Solid lipid nanoparticles (SLN) between 50 and 1000 nm are popular in cosmetics and drugs. SLNs exhibit UV-resistant qualities and can be used as a carrier for 3,4,5-trimethoxybenzoylchitin and vitamin E [58] to protect hair from UV rays. Biocompatibility and minimal toxicity toward cells [211,212,213] and organisms [214,215] make halloysite nanotubes suitable for daily haircare cosmetics [48]. Insecticide (permethrin) was placed into pure or hydrophobic halloysite to protect goats, capybaras, and guinea pigs against lice [216].

Ammonia, peroxide, p-phenylenediamine (PPD), diaminobenzene, toluene-2,5-diamine, resorcinol, etc., are commonly used dyes [217]. Permanent hair dyes are the most effective, but they can cause skin rashes, itching, hair loss, dandruff, irritation, cancer, asthma, allergic reactions, impaired eyesight, etc. [218]. Nanotechnology can minimize the negative effects of hair dyes. The aniline derivative p-phenylenediamine (PPD) is used in hair coloring and as a henna alternative. PPD-incorporated NPs were generated through the ion complex formation of PPD and poly(glutamic acid) (PGA). Glycol chitosan was added to reinforce PPD/PGA ion complexes. PPD-incorporated NPs attenuate PPD’s cytotoxic and allergenic effects [219]. 

Polydopamine (PDA) permits black hues (natural Asian hair colors) in human hairs in the presence of ferrous ions and involves three deposition mechanisms (i.e., polydopamine’s intrinsic binding capacity, metal-assisted self-assembly, and metal-related bridging between the keratin surface and polydopamine). Natural macromolecular molecules encapsulated in nanomaterials are noteworthy. *Bombyx mori* silk contains sericin and fibroin proteins. Sericin, a globular protein soluble in water with a molecular weight range of 10 to 300 kDa, has been added into conditioning products as cationic nanoparticles and has been shown to heal damaged cuticles and restore gloss and texture [196]. Sericin nanoparticles in hair dyes effectively protect color during washes. Dye formulations containing 3% sericin nanoparticles and a placebo mixture without nanoparticles have been applied to hair locks to evaluate the active agent’s coloring impact. The color was applied to the hair using hydrogen peroxide for 30 min. Then, the hair was cleaned and dried. The data and an examination of pictures of damaged hair treated with sericin nanoparticles demonstrated a return to a healthy appearance [197]. 

Gold nanoparticles (GNPs) synthesized inside the hair cortex were effectively employed to dye white hair a rich brown color that lasted for 16 days [198]. This study outlines the present state of knowledge about signaling pathways that are critical to the development and cycling of high-frequency hemoglobin. There is still much to learn about the signaling pathways and interactions that lead to fetal hair follicle development and morphogenesis, as well as the molecular distinctions that control fetal vs. postnatal hair follicle cycling. When it comes to treating androgenetic alopecia, telogen effluvium, alopecia, hirsutism, and wound healing and regeneration, the most important challenge will be translating insights from the biology of HFs into treatments for these and other conditions, including the de novo induction of HFs in adults. The proliferative and long-lived nature of HF stem cells raises the possibility of their acquiring and maintaining genetic alterations, which might eventually lead to tumor development. One reason for optimism that adult human hair follicle stem cells may soon be useful in regenerative therapies is the growing recognition that the hair follicle and the mesenchyme enveloping it are important sources of multipotent stem cell populations. Apart from what has been discussed above, new areas of hair study may soon alter our knowledge of hair follicle biology, such as comprehending the hair follicle bulb immunological privileges and the complicated endocrine functions. Recent formulations related to hair growth are tabulated in Table 1.

## 5. Biomedical Applications of Nanomaterials

The infection of lice by blood-feeding ectoparasitic insects of the order Phthiraptera is known as pediculosis. Every year, millions of children are infected with head lice, a condition known as pediculosis. Head lice have developed resistance to many of the presently used pediculicides, and insecticides and acaricides are harmful toxic substances that should always be used with caution, as they may impair human and animal health, necessitating the development of new effective therapies [220]. *Momordica charantia* (Cucurbitaceae) is a tropical liana used for food and medicine. *M. charantia* is antibacterial, antihelmintic, and antimycobacterial. An aqueous leaf extract was mixed with ZnO NPs to test their anti-parasitic efficacy. A SEM scan showed 21.32 nm spherical nanoparticles. GC-MS analysis showed that the M. charantia leaf extract contained the insect pheromone Nonacosane. Ticks, head lice, and mosquito larvae were treated with ZnO NPs and leaf extract for 24 h. Synthesized ZnO NPs in conjunction with M. charantia extract had good anti-parasitic action against *Pediculushumanus capitis*, *Anopheles stephensi*, *Culexquinquefasciatus*, and *Rhipicephalus (Boophilus) microplus* [221]. Lawsonia inermis plant extract used to develop silver nanoparticles (Ag NPs) was tested against human head lice (Pediculushumanus capitis De Geer (Phthiraptera: Pediculidae)) and sheep body lice (Bovicolaovis Schrank (Phthiraptera: Trichodectidae)). To investigate the pediculocidal activity of synthesized Ag NPs against B. ovis, the contact and impregnated methods were utilized with minor changes to increase practicality and efficiency. The synthesized Ag NPs were extremely stable and exhibited considerable adulticidal action against P. humanus capitis and B. ovis [222]. 

Chitosan, a chitin derivative, is utilized in cosmetics and biomedicine. Chitosan is biodegradable and has minimal immunogenicity [223]. Its usefulness in hair care was shown in a research study, boosting hair development in alopecia patients [224].

## 6. Nanoformulations for Hair Follicles (HF)

Currently available drugs cannot address non-cosmetic concerns such as hair loss. Hair follicles are a therapeutic target for regulating hair development [225]. The hair follicle is one avenue for drug transportation through the skin [226]. The degree of substance penetration relies on the density of follicles in each region of the skin. Hair follicles distribute topically administered chemicals. Lipid-based nanocarriers can be used for HF drug delivery and treatment of alopecia. Due to their chemical composition, lipid carriers offer an advantage for HF targeting and have been explored extensively [227]. Nanostructured lipid carriers (NLC), solid lipid nanoparticles (SLN) [201], liposomes [202], transferosomes [228], niosomes [229], and ethosomes [230] have been created to enhance the skin permeability of active compounds [231]. Smaller nanoparticles reveal larger depths of penetration by accumulating in hair follicles, facilitating medication administration at the capillary bulb. Particles of 200 nm size are better for medication delivery to the hair follicle isthmus [232]. When particle size reduces, surface area and dissolution rate increase, impacting the pharmacokinetic profile (accumulation area, release and distribution, metabolic transformation, etc.). Solid lipid nanoparticles containing minoxidil showed superior skin accumulation compared with commercial solutions [233]. The chemical structure of nano lipid carriers is important for drug distribution. NLC were created to overcome several possible limitations of SLN, such as its poor loading capacity and particle stability due to its high water content. SLN forms crystalline networks [100], causing drug leakage [234]. Wang et al. (2017) developed minoxidil-loaded NLC and SLN for topical alopecia therapy. MXD-NLC displayed a more prominent penetration and retention profile than MXD-SLN, minoxidil was released more quickly from NLC than SLN, and skin irritation tests revealed no erythema [201]. NLCs were loaded with clobetasol propionate in another investigation [235]. Finasteride-loaded NLCs have high physical and chemical storage stability [236] due to oleic acid. Oleic acid promotes an amorphous form in the solid lipid matrix, which lowers particle crystallinity and results in a high encapsulation efficiency [237]. 

Increased skin and tissue permeability improves liposome-based delivery methods. Liposomes produce a phospholipid coating on the skin that interacts with sebum to distribute drugs follicularly. Finasteride-loaded vesicular systems in 2% *w*/*w* methyl cellulose gel showed greater FNS penetration through excised abdominal mouse skin than an equivalent solution and traditional gels. Liposomal FNS formulations lasted 2 months refrigerated [202]. 

Transferosomes have a greater hydrophilicity than liposomes and are more resistant to fusing with skin lipids. Surfactants act as edge activators to provide lipid bilayer flexibility [238]. Transferosomes may transmit large molecules non-occlusively over intact mammalian skin. In vitro and in vivo, transfersomes have been employed to transport peptides, vaccines, anticancer medicines, and tiny medicinal compounds. In vivo investigations revealed that mice treated with a minoxidil and caffeine transferosome formulation increased their hair length and weight [239]. 

Squarticles, a lipid nano emulsion made from sebum-derived lipids such as squalene and fatty esters, were explored as a nanotechnology-based formulation for topical administration of minoxidil (a drug for hair growth promotion). The average diameter of cationic squarticles is greater than that of anionic squarticles. The lipidic structure of squarticles and their nano-size enhance interactions and fusion with sebum, allowing for targeted administration of minoxidil into follicles [240]. The benefits of NE were revealed in a comparison study of two separate nanosystems: nanostructured lipid carriers (NLC) and nano emulsions (NE) with Nile red. The highly homogenous distribution of red fluorescence over the treated skin was aided by NE containing Nile red. The stratum corneum and follicular ducts both have squarticles in the shape of NE. In the case of NE-treated skin, there was a higher intensity of Nile red fluorescence [241].

Gelatin, alginate [198], chitosan [199,200], albumin [242], poly(caprolactone) [201], poly(lactide-co-glycolide) copolymers, poly(amino acids), poly(lactide), and polymethacrylates [243] were used to make biodegradable and biocompatible polymeric nanoparticles for the treatment of alopecia. Polymeric nanoparticles can prevent encapsulated pharmaceuticals from degradation for several months [244], promote medication distribution consistency, and regulate drug release [199,245]. The relaxation of polymer chains or polymer breakdown (for example, because of fermentative hydrolysis in biological systems) leads to the release of drugs from carriers, which is the mechanism of drug release from these polymeric nanoparticles. Furthermore, during medication release, the aqueous phase interacts with the particles, causing the polymeric wall to relax. Polymer nanoparticles’ pharmacodynamics and tissue penetration depth are influenced by their size and chemical structure, just as lipid nanoparticles are. Smaller polymer nanoparticles (NPs) may penetrate deeper into hair follicles (HFs), whereas NPs larger than 5 m aggregate in the infundibulum of the HF [246]. Halloysite nanotubes [217], lipid nanocarriers, and nanocrystals [247] can be used to carry drugs that are poorly water soluble. Nanocrystals are a viable drug delivery strategy not only for oral and topical use but also for hair follicle targeting due to their higher kinetic solubility, which aids passive penetration through the skin and other particular features. 

Curcumin nanosuspensions (nanocrystals with a size of approximately 300 nm) were integrated into several gel bases (polar, non-polar hydrogels, and oleogels) to create gels with a curcumin content of 1% (*w*/*w*). The efficiency of hair follicle penetration, as well as passive skin penetration, was studied using an ex vivo pig ear model. The hair follicles were able to absorb nanocrystals that reached the lower region of the infundibulum as a result. The maximum amount of passive penetration was achieved by humectant-containing hydrogels; however, these hydrogels entered the hair follicles with a reduced efficiency [248].

## 7. Conclusions

In recent years, research on traditional herbal treatments has gained importance due to the lack of highly effective and safe products that can be used for hair growth promotion and hair loss treatment. The multiple effects of plant-based chemicals, especially the fact that they have been tried for many years with traditional use, and the continued application of those with low side effects has increased the orientation toward herbal resources. The formulation of plant-derived extracts or isolated chemicals with nanocarriers to improve efficacy, safety, and stability is promising. Considering these studies, it will be promising to carry out extensive studies with nanocarriers for hair growth promotion and hair loss treatment.

## Figures and Tables

**Table 1:** Hair growth formulations.

Sl No.	Plant	Formulation	Source	Mechansimof Action	References
1	Amla or Indian gooseberry	Fruit juice, capsule, and hair oil	*Emblica officinalis* linn. Family—euphorbiaceae	Powerful inhibitor of 5-alpha reductase	[107]
2	Brahmi, water hyssop	Capsule, booti, and powder	*Bacopa monnieri* linn. Family—plantaginaceae	Metal chelation at the initiation level and also as a chain breaker	[108]
3	Tapaswani	Capsule, powder, and tablet	*Nardostachys jatamansi* Family—caprifoliaceae	Increases the expression of hair growth factor	[109]
4	Fenugreek	Hair oil, capsule, and powder	*Trigonella foenum graecum* Family—fabaceae	Intervening in the anagen-to-catagen and catagen-to-telogen stages of the hair cycle	[110]
5	Rose mallow, China rose, and shoeblack plant	Paste of fresh or dry flower extract	*Hibiscus rosa*-sinensis linn. Family—malvaceae	Improve the build-up of keratin and boost the formation of new hair follicles	[111]
6	Umbrella polypore, lumpy bracket	Capsule, power, and extract	*Polyporus umbellatus* Family—polyporaceae	Inhibiting catagen entry in the human hair follicle organ	[112]
7	Rosemary	Oil and tincture	*Rosmarinus officinalis* linn. Family—lamiaceae	Blockage of DHT precursor, 5-alpha-reductase	[113]
8	Daisy and aster	Hair oil, tablet	*Arnica montana* Family—asteraceae	Increases blood circulation in the scalp and hair follicles	[114]
9	Ramie	Oil	*Boehmeria nipononivea* Family—urticaceae	Short t telogen, causing premature entry of resting hair follicles	[115]
10	Himalayan boxwood	Fresh or dry bark extract	*Buxus wallichiana* baill Family—buxaceae	5-alpha reductase inhibition	[116]
11	Maidenhair tree	Fresh or dry fruit and leaf extract	*Ginkgo biloba* tree linn. Family—ginkgoaceae	Improves circulation in the scalp	[117]
12	Brahmi	Hair cleanser, oil, powder, fresh leaves	*Centella asiatica* Family—umbelliferae	Makes the follicles and roots stronger, helping better and stronger hair to grow back	[118]
13	Bhringraj	Oil, hair tonic	*Eclipta alba* linn. Family—asteraceae	Increases blood circulation to the scalp and roots	[119]
14	Coconut	Oil, shampoos, serum	*Cocos nucifera* linn. Family—palmae	The vitamins and essential fatty acids naturally found in coconut oil nourish the scalp and help to remove sebum build-up from hair follicles.	[120]
15	*Ginseng radix*	Powder, capsule, serum	*Panax ginseng* Family—araliaceae	Prevent apoptosis of hair follicle cells and inhibit 5-α reductase	[121]
16	Sage oil	Oil, tincture	*Salvia officinalis* linn. Family—labiatae	Improve blood circulation to the scalp	[122]
17	Holy basil oil, tulsi	Powder, oil, tincture, leaf and seed extracts	*Ocimum sanctum* Family—lamiaceae	Increases blood flow and makes the hair root healthy	[123]
18	Jojoba oil	Oil	*Simmondsia chinensis* Family—simmondiaceae	Stimulates circulation in the scalp, nourishing and strengthening the hair follicles to grow	[124]
19	Japanese fern spores	Oil, powder	Climbing greenery Family—lygodiaceae	5-alpha reductase inhibition	[125]
20	Ghamra and coatbutton	Oil	*Tridax procumbens* linn. Family—daisy	Unknown	[126]
21	Indian subcontinen	Oil	*Cuscuta reflexa* roxb Family—convolvulaceae	Unknown	[127]
22	Onion	Oil, shampoo	*Allium cepa* l Family—liliaceae	Onion juice can provide extra sulfur to support strong and thick hair, thus preventing hair loss and promoting hair growth	[128]
23	Tuber fleeceflower	Oil, tonic	*Polygonium multiflorum* thumb Family—polygonaceae		[129]
24	Peppers, capsicums	Powder, oil, and extract	*Capsicum annum* linn. Family—solanaceae	PI3K/AKT pathway	[130]
25	Northern white cedar	Oil, serum	*Thujae occidentalis* semen Family—cupressaceae	Enhances circulation to the scalp	[131]
26	Grape seeds and blueberry	Oil, shampoo, capsule	Proanthocyanidin class of flavonoids Family—sulfotransferase	Catalyze the release of histamine	[132]
27	Green tea	Oil, shampoo, paste, gel, and serum	*Camellia sinensis* Family—theaceae	Selectively inhibiting 5-alpha reductase	[133]

**Table 2:** Patents for hair growth promotion.

Sl No.	Patent No	Formulation	Route	Excipient	Mechanism of Action	References
1	US8957112	Cream gel	Dermatology	Dibenzoyl peroxide	Benzoyl peroxide exhibits bactericidal effects against Cutibacterium *acnes,* a key component of acne vulgaris.	[134]
2	CN10126973 B	Shampoo	Subcutaneous	Reynoutria multiflora, plastycladus orientalis, Notopterygium forbesii Boiss, dried root of Angelica sinensis, erial parts of *Eclipta* prostrata L., pericarpium zanthoxyli, *Menthae* Haplocalycis *Herba*, *Zingiberis Rhizoma* Recens	Bactericidal	[135]
3	EP2421498 A1	Conditioner	Topical	6-Benzyladenine, mixtures of the esters of these fatty acids with the polyglycerol mixture, fatty acid esters	Bactericidal	[136]
4	US20120165291	Cosmetic preparation	Topical	Azole antifungals and zinc salt of gluconic acid	Promote DNA and RNA production.	[137]
5	WO2012080223 A1	Film	Topical	Glycosaminoglycan alkoxy, formic acid, α-lipoic acid	Bactericidal	[138]
6	US20120165291 A1	Viscous aqueous solution	Topical	Polysaccharide(Acidic) and water	Increase the contents of VEGF and HGF in the skin tissue of alopecia areata	[139]
7	DE102010015120 B4	Cosmetic preparation	Topical	Trypsin, glycosylated, Ca^2+^, and chymotrypsin	Bactericidal	[140]
8	CN102724959 B	Oil	Topical	Extract of duchesneae indicae	Elongate the anagen phase and abrogate the effects of androgen	[141]
9	WO2013148377 A1	Micro needle device	Topical	Laminin-511	Laminin-511 promoted hair growth through morphogenic signaling, resulting in Shh and noggin expression	[142]
10	CA2489308 C	Tablet	Oral	Taurine	Flushes toxins from the scalp, removing excess sebum as well as dead skin cells and DHT	[143]
11	WO2014041140 A1	Pharmaceutical salt	Epidermal	3-(4-aminophenyl)-2-methoxypropanoic acid	Bactericidal	[144]
12	US20140065086	Cosmetic preparation	Topical	DICKKOPF-1	Attenuation of the hair growth process by inhibiting Wnt/β-catenin signaling via the LRP5/6 co-receptor	[145].
13	CN103520048 A	Shampoo	Topical	Polyquaternium-10, Chinese herb medication, ammonium lauryl sulfate	Inhibits the 5-alpha reductase enzyme	[146]
14	EP2674148 A1	Hair tonic	Topical	Boat orchids (Genus cymbidium)	Restriction of fungal growth	[147]
15	US8603545	Cosmetic	Topical	Genus buchholzia	Induces the anagen phase in resting hair follicles	[148]
16	WO2013180229 A1	Hair tonic	Topical	Group of alkoxycarbonyls	Stimulates hair growth, is not fully understood	[149]
17	WO2013167927 A1	Injectable	Inj.	Biotin, organic silicon, minerals, pentoxyfilline, and hydrochloride salt form of procaine	Enhances keratin production	[150]
18	EP2162115 B1	Balm, lotion, emulsion, paste, tablet, cream, foam or spray, particularly in emulsified form	Oral and topical	Advanced glycation end products	Unknown	[151]
19	CN103445997 A	Shampoo	Topical	*Cacumen biotae*, root of Polygonum, lycii radicis cortex, *Curcuma longa*, deionized water, lauryl sodium sulphate (SLS), sodium alkylethersulfate, *Alkyl* polyglycosides, emulsified silicone, *Cocamidopropyl betaine* (CAPB), hexadecyl alcohol, stearyl polyoxyethyl hydroxyethyl ammonium chloride, ethylene glycol stearate diester, ammonium chloride, chitin polysaccharide, essence, kathon, acrylamide methyl ammonium oxide, guar gum	Anti-fungal	[152]
20	US20140171496 A1	Moisturizer	Topical	Prostaglandin compound	Stimulation of hair follicle stem cells by prostaglandin E2 collagen matrix	[153]
21	US20140170246 A1	Foam	Topical	Depilatory agent	Disrupts the disulfide bonds of hair keratin	[154]
22	EP2740741 A1	Cosmetics	Topical	Acetyl group, fluorenyl methoxy carbonyl group, formyl group, palmitoyl group, myristyl group, stearyl group, and polyethylene glycol (peg)	Promotes melanin production	[155]
23	US8962041 B2	Cosmetics	Topical	Blackberry	Increases blood flow to scalp	[156]
24	WO2015012198 A1	Moisturizer	Topical	Kluyveromyces and a polyhydric alcohol	Unknown	[157]

**Table 3:** Clinical studies for the evaluation of hair growth formulations.

Cell Studies for Hair Growth	Composition	Results	References
Minoxidil—topical (5% *w/v*)	Minoxidil (50 mg/mL) propyleneglycol (500 mg/mL), ethanol (300 mg/mL), water	Three patients who used a 5% minoxidil solution for a year saw some hair regrowth. Two of these three individuals developed barely discernible, tiny, pigmented terminal hairs in scalp locations that had previously had only vellus hairs. Hair regrowth occurred after 4 and 20 weeks, respectively. The third patient exhibited a noticeable restoration of bigger, thicker, more pigmented terminal hair.	[158]
Finasteride (0.5% topical solution)	Finasteride	There was an observable increase in hair count (baseline = 876 hairs) with finasteride treatment, measured in a 1-inch diameter circular area of balding vertex scalp. Self-assessment of patients confirmed that there was a decrease in the rate of hair loss with an increase in the growth of hair with finasteridetreatment.	[159]
Intralesional triamcinolone acetonide injection	Triamcinolone acetonide	A total of 3 injections of triamcinolone acetonide 2.5 mg/mL were given at an interval of 3 weeks to the patients. The results showed that, after follow-up, more than 50% hair regrowth was observed in 27 (67.5%) patients with intralesional steroids at the end of the treatment.	[160]
Spironolactone (systemic)	Spironolactone	Spironolactone at a dose of 200 mg daily was observed to reduce loss of hair by 50–62.9% in a case study of 4 patients. It has also been observed that there is an increase in the total number of anagen hairs.	[161]
Dutasteride (oral)	Dutasteride	Dutasteride was seen to increase the mean hair counts by 12.2/cm^2^ compared with 4.7/cm^2^ in the placebo group in the treatment of MPHL.	[162]
Valproic acid	Valproic acid	Forty male patients with moderate AGA were involved in a study. They received treatment with either VPA (sodium valproate, 8.3%) or placebo spray for 24 weeks. Twenty-seven out of forty patients (*n* = 15, VPA group; *n* = 12, placebo group) completed the whole protocol with good compliance. The mean change in total hair count was seen to be significantly increased in the VPA group compared with the placebo group.	[163]
Flutamide (topical)	Flutamide	According to a study, flutamide at a dose of 250 mg daily showed an improvement in the growth of hair compared with 5 mg of finasteride daily and 50 mg of cyproterone acetate daily. Flutamide showed a reduction of 21% in Ludwig scores compared with the other two drugs.	[164]
Saw Palmetto (topical)	Contains fatty acids (85–90%), carotenoids, lipases, tannins, and sugars, as well as beta-sitosterol, anthranilic acid, capric acid, caproic acid, caprylic acid, carotene, ferulic acid, linoleic acid, myristic acid, lauric acid, oleic acid, palmitic acid, 1-monolaurin, and 1-mono-myristin	In a 2012 study involving 100 males taking 320 milligrams (mg) of saw palmetto each day over 2 years, it was seen that 38% of those who took saw palmetto had improvements in their hair loss.	[165]
Ketoconazole (topical)	Ketoconazole	2% Ketoconazole shampoo on MPHL has been observed to increase hair density along with increasing the size and proportion of anagen follicles.	[166]
Green tea (topical)	Antioxidants such as polyphenols and flavonoids that contain catechins and their derivatives, epicatechin (EC), epigallocatechin gallate (EGCG), epigallo catechins, epicatechin gallate, linoleic and linolenic acids, and vitamins		[167]
Pumpkin seed	Polyunsaturated fatty acids of 80% palmitic acid, myristic acid, stearic acid, oleic acid, and linoleic acid, as well as vitamin E, α-tocopherols, γ-tocopherols, carotenoid, phytoestrogens, and phytosterols		[168]
Rosemary oil (topical)	Contains esters (2.6%) largely as borneol, cineoles, and several terpenes, chiefly a-pinene, camphene, 1% and 2% volatile oil containing 0.8% and 6% esters, and 8% and 20% alcohols, respectively		[169]
Grapeseed oil (topical)	Anthocyanins, flavan-3-ols (example: catechins), vitamin-E (α-tocopherol), petiole, linoleic acid, flavonoids (resveratrol, quercetin and catechin, and polyphenols (flavonoids, phenolic acids, phenolic alcohols, stilbenes, and lignans), and trimer gallate, unsaturated fatty acids, and phytosterols		[170]
Licorice (topical)	Glycyrrhetinic acids rich in flavonoids such as liquiritin, isoliquiritin, neoisoliquiritin, liquiritigenin, glycerin, glyzaglabrin, and licoisofavines.		[171]
Tinfal Plus Serum (topical)	Minoxidil 5% + Aminexil 1.5%		[172]
Keraglo Eva (topical)	Biotin (10 Mg), folic acid (300 Mcg), selenium (40 Mcg) (173)		[173]
**Organ model studies**			
Human hair follicle (HF) unit		The anagen–catagen transition in organ-cultured, severed human scalp HFs differs from the transformation in vivo in a number of ways, including the absence of the bulge, isthmus area, sebaceous gland, and tissue interactions with the dermis and subcutis. As a result, it has long been unclear to what degree in vivo morphological criteria may be transferred to HF organ culture settings. This article ma an effort to address these technical issues.	[174]
Rat vibrissae follicle unit		The histopathology of the proximal follicle bulb revealed that vibrissa follicles extracted from 12-day-old rats were in the anagen stage of their hair development cycle. The extended dermal papilla (DP) was located inside the follicular bulb and was surrounded by highly basophilic epithelial matrix cells, which displayed typical patterns of lineage-restricted differentiation, giving rise to the keratinized hair fiber and inner root sheath.	[175]
Full-thickness human scalp skin			
Animal Models			
Anagen phase induction models		The anagen–catagen transition in severed human scalp organ culture HFs differs from this metamorphosis in vivo in a number of ways, including the absence of the bulge, isthmus area, sebaceous gland, and tissue interactions with the dermis and subcutis. As a result, it has long been unclear to what degree in vivo morphological criteria may be transferred to HF organ culture settings. This article made an effort to address these technical issues.	[176]
Androgen effect modulation models			
Mesocricetus auratus (golden hamster)		This model was used to assess macroscopic and microscopic evaluation (hair diameter analysis) as an animal model for hair regrowth.	[177]
C3 H mouse model		Even though the increase in density of hair of the animal and the wave pattern hair cycle development provided drawbacks, they were the most extensively reported for hair growth promotion. After just two weeks of treatment, laser therapy administered to C3 H mice for 20 s daily, three times per week, caused a substantially longer development phase, with the majority of the follicles from the examined region in the anagen hair growth phase.	[178]
Progenitor cell population in mice		These cells are comparable to human cells. These mature cells were tested on immunodeficient mice animal models, and the findings demonstrated the creation of new hair follicles and enhanced hair regrowth.	[179]

## Data Availability

All data available within the manuscript.

## References

[1] Hamilton J.B. (1951). Patterned loss of hair in man: Types and incidence. Ann. N. Y. Acad. Sci..

[2] Shankar D.K., Chakravarthi M., Shilpakar R. (2009). Male androgenetic alopecia: Population-based study in 1005 subjects. Int. J. Trichol..

[3] Birch M., Messenger J., Messenger A. (2001). Hair density, hair diameter and the prevalence of female pattern hair loss. Br. J. Dermatol..

[4] Norwood O.T.T. (2001). Incidence of female androgenetic alopecia (female pattern alopecia). Dermatol. Surg..

[5] Lolli F., Pallotti F., Rossi A., Fortuna M.C., Caro G., Lenzi A., Sansone A., Lombardo F. (2017). Androgenetic alopecia: A review. Endocrine.

[6] Mahe Y.F., Michelet J.-F., Billoni N., Jarrousse F., Buan B., Commo S., Saint-Leger D., Bernard B.A. (2000). Androgenetic alopecia and microinflammation. Int. J. Dermatol..

[7] Magro C.M., Rossi A., Poe J., Manhas-Bhutani S., Sadick N. (2011). The role of inflammation and immunity in the pathogenesis of andro-genetic alopecia. J. Drugs Dermatol. JDD.

[8] Martinez-Jacobo L., Ancer-Arellano C.I., Ortiz-Lopez R., Salinas-Santander M., Villarreal-Villarreal C.D., Ancer-Rodriguez J., Camacho-Zamora B., Zomosa-Signoret V., La Garza C.E.M.-D., Ocampo-Candiani J. (2017). Evaluation of the Expression of Genes Associated with Inflammation and Apoptosis in Androgenetic Alopecia by Targeted RNA-Seq. Ski. Appendage Disord..

[9] Dey-Rao R., Sinha A. (2017). A genomic approach to susceptibility and pathogenesis leads to identifying potential novel therapeutic targets in androgenetic alopecia. Genomics.

[10] Upton J.H., Hannen R.F., Bahta A.W., Farjo N., Farjo B., Philpott M.P. (2015). Oxidative Stress–Associated Senescence in Dermal Papilla Cells of Men with Androgenetic Alopecia. J. Investig. Dermatol..

[11] Prie B., Iosif L., Tivig I., Stoian I., Giurcaneanu C. (2016). Oxidative stress in androgenetic alopecia. J. Med. Life.

[12] Hibino T., Nishiyama T. (2004). Role of TGF-β2 in the human hair cycle. J. Dermatol. Sci..

[13] Kwack M.H., Ahn J.S., Kim M.K., Kim J.C., Sung Y.K. (2012). Dihydrotestosterone-Inducible IL-6 Inhibits Elongation of Human Hair Shafts by Suppressing Matrix Cell Proliferation and Promotes Regression of Hair Follicles in Mice. J. Investig. Dermatol..

[14] Titeca G., Goudetsidis L., Francq B., Sampogna F., Gieler U., Tomas-Aragones L., Lien L., Jemec G.B.E., Misery L., Szabo C. (2020). ‘The psychosocial burden of alopecia areata and androgenetica’: A cross-sectional multicentre study among dermatological out-patients in 13 European countries. J. Eur. Acad. Dermatol. Venereol..

[15] Goren A., Shapiro J., Roberts J., Desai N., Zarrab Z., Pietrzak A., Lotti T. (2015). Clinical utility and validity of minoxidil response testing in androgenetic alopecia. Dermatol. Ther..

[16] Lee S.W., Juhasz M., Mobasher P., Ekelem C., Mesinkovska N.A. (2018). A systematic review of topical finasteride in the treatment of androgenetic alopecia in men and women. J. Drugs Dermatol. JDD.

[17] Monti D., Tampucci S., Burgalassi S., Chetoni P., Lenzi C., Pirone A., Mailland F. (2014). Topical Formulations Containing Finasteride. Part I: In Vitro Permeation/Penetration Study and In Vivo Pharmacokinetics in Hairless Rat. J. Pharm. Sci..

[18] Olsen E.A., Whiting D., Bergfeld W., Miller J., Hordinsky M., Wanser R., Zhang P., Kohut B. (2007). A multicenter, randomized, placebo-controlled, double-blind clinical trial of a novel formulation of 5% minoxidil topical foam versus placebo in the treatment of androgenetic alopecia in men. J. Am. Acad. Dermatol..

[19] Price V.H., Roberts J.L., Hordinsky M., Olsen E.A., Savin R., Bergfeld W., Fiedler V., Lucky A., Whiting D.A., Pappas F. (2000). Lack of efficacy of finasteride in postmenopausal women with androgenetic alopecia. J. Am. Acad. Dermatol..

[20] Whiting D.A., Olsen E.A., Savin R., Halper L., Rodgers A., Wang L., Hustad C., Palmisano J. (2003). Efficacy and tolerability of finasteride 1 mg in men aged 41 to 60 years with male pattern hair loss. Eur. J. Dermatol..

[21] Friedman E.S., Friedman P.M., Cohen D.E., Washenik K. (2002). Allergic contact dermatitis to topical minoxidil solution: Etiology and treatment. J. Am. Acad. Dermatol..

[22] Lawson C.N., Hollinger J., Sethi S., Rodney I., Sarkar R., Dlova N., Callender V.D. (2017). Updates in the understanding and treatments of skin & hair disorders in women of color. Int. J. Women’s Dermatol..

[23] Khumalo N., Jessop S., Gumedze F., Ehrlich R. (2007). Hairdressing and the prevalence of scalp disease in African adults. Br. J. Dermatol..

[24] Samrao A., Price V.H., Zedek D., Mirmirani P. (2011). The “Fringe Sign”—A useful clinical finding in traction alopecia of the marginal hair line. Dermatol. Online J..

[25] Uwakwe L., De Souza B., Tovar-Garza A., McMichael A. (2020). Intralesional Triamcinolone Acetonide in the Treatment of Traction Alopecia. J. Drugs Dermatol..

[26] Callender V.D., McMichael A.J., Cohen G.F. (2004). Medical and surgical therapies for alopecias in black women. Dermatol. Ther..

[27] Khumalo N., Ngwanya R. (2007). Traction alopecia: 2% topical minoxidil shows promise. Report of two cases. J. Eur. Acad. Dermatol. Venereol. JEADV.

[28] Ogunleye T.A., McMichael A., Olsen E.A. (2014). Central centrifugal cicatricial alopecia: What has been achieved, current clues for future research. Dermatol. Clin..

[29] Malki L., Sarig O., Romano M.-T., Méchin M.-C., Peled A., Pavlovsky M., Warshauer E., Samuelov L., Uwakwe L., Briskin V. (2019). Variant *PADI3* in Central Centrifugal Cicatricial Alopecia. N. Engl. J. Med..

[30] Dinh Q.Q., Sinclair R. (2007). Female pattern hair loss: Current treatment concepts. Clin. Interv. Aging.

[31] Roe A.L., McMillan D.A., Mahony C. (2018). A Tiered Approach for the Evaluation of the Safety of Botanicals Used as Dietary Supplements: An Industry Strategy. Clin. Pharmacol. Ther..

[32] Schneider M.R., Schmidt-Ullrich R., Paus R. (2009). The Hair Follicle as a Dynamic Miniorgan. Curr. Biol..

[33] Yang C.-C., Cotsarelis G. (2010). Review of hair follicle dermal cells. J. Dermatol. Sci..

[34] Perrimon N., Pitsouli C., Shilo B.-Z. (2012). Signaling Mechanisms Controlling Cell Fate and Embryonic Patterning. Cold Spring Harb. Perspect. Biol..

[35] Woo W.-M., Zhen H.H., Oro A.E. (2012). Shh maintains dermal papilla identity and hair morphogenesis via a Noggin—Shh regulatory loop. Genes Dev..

[36] Kumari S., Goyal A., Garg M., Antonescu A., Sindhu R.K. (2023). Lyotropic Liquid Crystal System for Drug Delivery of Astaxanthin: Physical Characterization and Enhanced Antioxidant Potential. Crystals.

[37] Rishikaysh P., Dev K., Diaz D., Qureshi W.M.S., Filip S., Mokry J. (2014). Signaling Involved in Hair Follicle Morphogenesis and Development. Int. J. Mol. Sci..

[38] Oh J.W., Kloepper J., Langan E.A., Kim Y., Yeo J., Kim M.J., Hsi T.-C., Rose C., Yoon G.S., Lee S.-J. (2016). A Guide to Studying Human Hair Follicle Cycling In Vivo. J. Investig. Dermatol..

[39] Singh S., Sindhu R.K., Alsayegh A.A., Batiha G.E., Alotaibi S.S., Albogami S.M., Conte-Junior C.A. (2023). Formulation Development and Investigations on Therapeutic Potential of Nanogel from *Beta vulgaris* L. Extract in Testosterone-Induced Alopecia. BioMed Res. Int..

[40] Ohyama M., Terunuma A., Tock C.L., Radonovich M.F., Pise-Masison C.A., Hopping S.B., Brady J.N., Udey M.C., Vogel J.C. (2005). Characterization and isolation of stem cell-enriched human hair follicle bulge cells. J. Clin. Investig..

[41] Plikus M.V., Chuong C.-M. (2008). Complex Hair Cycle Domain Patterns and Regenerative Hair Waves in Living Rodents. J. Investig. Dermatol..

[42] Tobin D.J. (2009). Aging of the hair follicle pigmentation system. Int. J. Trichol..

[43] Mesler A.L., Veniaminova N.A., Lull M.V., Wong S.Y. (2017). Hair Follicle Terminal Differentiation Is Orchestrated by Distinct Early and Late Matrix Progenitors. Cell Rep..

[44] Kruglikov I.L., Scherer P.E. (2016). Dermal adipocytes and hair cycling: Is spatial heterogeneity a characteristic feature of the dermal adipose tissue depot?. Exp. Dermatol..

[45] Geyfman M., Plikus M.V., Treffeisen E., Andersen B., Paus R. (2014). Resting no more: Re-defining telogen, the maintenance stage of the hair growth cycle. Biol. Rev..

[46] Lyle S., Elder D.E., Christofidou-Solomidou M., Liu Y., Albelda S., Cotsarelis G. (1999). Human Hair Follicle Bulge Cells are Biochemically Distinct and Possess an Epithelial Stem Cell Phenotype. J. Investig. Dermatol. Symp. Proc..

[47] Mistriotis P., Andreadis S.T. (2013). Hair follicle: A novel source of multipotent stem cells for tissue engineering and regenerative medicine. Tissue Eng. Part B Rev..

[48] Roh C., Tao Q., Photopoulos C., Lyle S. (2005). In Vitro Differences Between Keratinocyte Stem Cells and Transit-Amplifying Cells of the Human Hair Follicle. J. Investig. Dermatol..

[49] Martel J., Badri T. (2018). Anatomy, Head, Hair, Follicle.

[50] Hsu Y.-C., Pasolli H.A., Fuchs E. (2011). Dynamics between Stem Cells, Niche, and Progeny in the Hair Follicle. Cell.

[51] Plikus M.V. (2012). New Activators and Inhibitors in the Hair Cycle Clock: Targeting Stem Cells’ State of Competence. J. Investig. Dermatol..

[52] Burg D., Yamamoto M., Namekata M., Haklani J., Koike K., Halasz M. (2017). Promotion of anagen, increased hair density and reduction of hair fall in a clinical setting following identification of FGF5-inhibiting compounds via a novel 2-stage process. Clin. Cosmet. Investig. Dermatol..

[53] Rodriguez C.N., Nguyen H. (2017). Identifying Quiescent Stem Cells in Hair Follicles. Cell. Quiescence Methods Protoc..

[54] Higgins C.A., Westgate G.E., Jahoda C.A. (2009). From Telogen to Exogen: Mechanisms Underlying Formation and Subsequent Loss of the Hair Club Fiber. J. Investig. Dermatol..

[55] Wang K., Li M., Hakonarson H. (2010). ANNOVAR: Functional annotation of genetic variants from high-throughput sequencing data. Nucleic Acids Res..

[56] Erdoğan B. (2017). Anatomy and Physiology of Hair. Hair and Scalp Disorders.

[57] Harrison S., Sinclair R. (2002). Telogen effluvium. Clin. Exp. Dermatol. Clin. Dermatol..

[58] Soteriou D., Kostic L., Sedov E., Yosefzon Y., Steller H., Fuchs Y. (2016). Isolating hair follicle stem cells and epidermal keratinocytes from dorsal mouse skin. J. Vis. Exp..

[59] Cotsarelis G., Sun T.-T., Lavker R.M. (1990). Label-retaining cells reside in the bulge area of pilosebaceous unit: Implications for follicular stem cells, hair cycle, and skin carcinogenesis. Cell.

[60] Lyle S., Christofidou-Solomidou M., Liu Y., Elder D.E., Albelda S., Cotsarelis G. (1998). The C8/144B monoclonal antibody recognizes cytokeratin 15 and defines the location of human hair follicle stem cells. J. Cell Sci..

[61] Oshima H., Rochat A., Kedzia C., Kobayashi K., Barrandon Y. (2001). Morphogenesis and Renewal of Hair Follicles from Adult Multipotent Stem Cells. Cell.

[62] Harries M.J., Paus R. (2010). The Pathogenesis of Primary Cicatricial Alopecias. Am. J. Pathol..

[63] Bose A., Teh M.-T., Mackenzie I.C., Waseem A. (2013). Keratin K15 as a Biomarker of Epidermal Stem Cells. Int. J. Mol. Sci..

[64] Rompolas P., Greco V. (2014). Stem cell dynamics in the hair follicle niche. Seminars in Cell & Developmental Biology.

[65] Myung P., Ito M. (2012). Dissecting the bulge in hair regeneration. J. Clin. Investig..

[66] Houschyar K.S., Momeni A., Pyles M.N., Maan Z.N., Whittam A.J., Siemers F. (2015). Wnt signaling induces epithelial differentiation during cutaneous wound healing. Organogenesis.

[67] Nakamura M., Schneider M.R., Schmidt-Ullrich R., Paus R. (2013). Mutant laboratory mice with abnormalities in hair follicle morphogenesis, cycling, and/or structure: An update. J. Dermatol. Sci..

[68] Leishman E., Howard J.M., Garcia G.E., Miao Q., Ku A.T., Dekker J.D., Tucker H., Nguyen H. (2013). Foxp1 maintains hair follicle stem cell quiescence through regulation of Fgf18. Development.

[69] Ohnemus U., Uenalan M., Conrad F., Handjiski B., Mecklenburg L., Nakamura M., Inzunza J., Gustafsson J.-A., Paus R. (2005). Hair Cycle Control by Estrogens: Catagen Induction via Estrogen Receptor (ER)-α Is Checked by ERβ Signaling. Endocrinology.

[70] Narhi K., Jarvinen E., Birchmeier W., Taketo M.M., Mikkola M.L., Thesleff I. (2008). Sustained epithelial β-catenin activity induces precocious hair development but disrupts hair follicle down-growth and hair shaft formation. Development.

[71] Kandyba E., Kobielak K. (2014). Wnt7b Is an Important Intrinsic Regulator of Hair Follicle Stem Cell Homeostasis and Hair Follicle Cycling. Stem Cells.

[72] Lien W.-H., Polak L., Lin M., Lay K., Zheng D., Fuchs E. (2014). In vivo transcriptional governance of hair follicle stem cells by canonical Wnt regulators. Nature.

[73] Sethi J.K., Vidal-Puig A. (2010). Wnt signalling and the control of cellular metabolism. Biochem. J..

[74] Kobielak K., Stokes N., de la Cruz J., Polak L., Fuchs E. (2007). Loss of a quiescent niche but not follicle stem cells in the absence of bone morphogenetic protein signaling. Proc. Natl. Acad. Sci. USA.

[75] Tumbar T., Guasch G., Greco V., Blanpain C., Lowry W.E., Rendl M., Fuchs E. (2004). Defining the Epithelial Stem Cell Niche in Skin. Science.

[76] Morris R.J., Liu Y., Marles L., Yang Z., Trempus C., Li S., Lin J., Sawicki J.A., Cotsarelis G. (2004). Capturing and profiling adult hair follicle stem cells. Nat. Biotechnol..

[77] Rajendran R.L., Gangadaran P., Bak S.S., Oh J.M., Kalimuthu S., Lee H.W., Baek S.H., Zhu L., Sung Y.K., Jeong S.Y. (2017). Extracellular vesicles derived from MSCs activates dermal papilla cell in vitro and promotes hair follicle conversion from telogen to anagen in mice. Sci. Rep..

[78] Blanpain C., Fuchs E. (2006). Epidermal Stem Cells of the Skin. Annu. Rev. Cell Dev. Biol..

[79] Horsley V., Aliprantis A.O., Polak L., Glimcher L.H., Fuchs E. (2008). NFATc1 Balances Quiescence and Proliferation of Skin Stem Cells. Cell.

[80] Kandyba E., Leung Y., Chen Y.-B., Widelitz R., Chuong C.-M., Kobielak K. (2013). Competitive balance of intrabulge BMP/Wnt signaling reveals a robust gene network ruling stem cell homeostasis and cyclic activation. Proc. Natl. Acad. Sci. USA.

[81] Samuelov L., Sprecher E., Tsuruta D., Bíró T., Kloepper J.E., Paus R. (2012). P-Cadherin Regulates Human Hair Growth and Cycling via Canonical Wnt Signaling and Transforming Growth Factor-β2. J. Investig. Dermatol..

[82] Woo W.-M., Oro A.E. (2011). SnapShot: Hair follicle stem cells. Cell.

[83] Greco V., Chen T., Rendl M., Schober M., Pasolli H.A., Stokes N., dela Cruz-Racelis J., Fuchs E. (2009). A Two-Step Mechanism for Stem Cell Activation during Hair Regeneration. Cell Stem Cell.

[84] Enshell-Seijffers D., Lindon C., Kashiwagi M., Morgan B.A. (2010). β-catenin Activity in the Dermal Papilla Regulates Morphogenesis and Regeneration of Hair. Dev. Cell.

[85] Wu H., Che X., Zheng Q., Wu A., Pan K., Shao A., Wu Q., Zhang J., Hong Y. (2014). Caspases: A Molecular Switch Node in the Crosstalk between Autophagy and Apoptosis. Int. J. Biol. Sci..

[86] Chuma M., Endo-Umeda K., Shimba S., Yamada S., Makishima M. (2012). Hairless Modulates Ligand-Dependent Activation of the Vitamin D Receptor-Retinoid X Receptor Heterodimer. Biol. Pharm. Bull..

[87] Teichert A., Elalieh H., Bikle D. (2010). Disruption of the hedgehog signaling pathway contributes to the hair follicle cycling deficiency in Vdr knockout mice. J. Cell Physiol..

[88] Tampucci S., Burgalassi S., Chetoni P., Lenzi C., Pirone A., Mailland F., Caserini M., Monti D. (2014). Topical Formulations Containing Finasteride. Part II: Determination of Finasteride Penetration into Hair Follicles using the Differential Stripping Technique. J. Pharm. Sci..

[89] Higgins C.A., Petukhova L., Harel S., Ho Y.Y., Drill E., Shapiro L., Wajid M., Christiano A.M. (2014). FGF5 is a crucial regulator of hair length in humans. Proc. Natl. Acad. Sci. USA.

[90] Foitzik K., Spexard T., Nakamura M., Halsner U., Paus R. (2005). Towards Dissecting the Pathogenesis of Retinoid-Induced Hair Loss: All-Trans Retinoic Acid Induces Premature Hair Follicle Regression (Catagen) by Upregulation of Transforming Growth Factor-β2 in the Dermal Papilla. J. Investig. Dermatol..

[91] Plikus M.V., Mayer J.A., de La Cruz D., Baker R.E., Maini P.K., Maxson R., Chuong C.M. (2008). Cyclic dermal BMP signalling regulates stem cell activation during hair regeneration. Nature.

[92] Castellana D., Paus R., Perez-Moreno M. (2014). Macrophages Contribute to the Cyclic Activation of Adult Hair Follicle Stem Cells. PLoS Biol..

[93] Kim M.-J., Choe S. (2011). BMPs and their clinical potentials. BMB Rep..

[94] Morgan B.A. (2014). The Dermal Papilla: An Instructive Niche for Epithelial Stem and Progenitor Cells in Development and Regeneration of the Hair Follicle. Cold Spring Harb. Perspect. Med..

[95] Mecklenburg L., Tobin D.J., Cirlan M.V., Craciun C., Paus R. (2005). Premature termination of hair follicle morphogenesis and accelerated hair follicle cycling in Iasi congenital atrichia (fzica) mice points to fuzzy as a key element of hair cycle control. Exp. Dermatol..

[96] Chueh S.-C., Lin S.-J., Chen C.-C., Lei M., Wang L.M., Widelitz R., Hughes M.W., Jiang T.-X., Chuong C.M. (2013). Therapeutic strategy for hair regeneration: Hair cycle activation, niche environment modulation, wound-induced follicle neogenesis, and stem cell engineering. Expert Opin. Biol. Ther..

[97] Garza L.A., Yang C.-C., Zhao T., Blatt H.B., Lee M., He H., Stanton D.C., Carrasco L., Spiegel J.H., Tobias J.W. (2011). Bald scalp in men with androgenetic alopecia retains hair follicle stem cells but lacks CD200-rich and CD34-positive hair follicle progenitor cells. J. Clin. Investig..

[98] Stenn K.S., Cotsarelis G. (2005). Bioengineering the hair follicle: Fringe benefits of stem cell technology. Curr. Opin. Biotechnol..

[99] Steinberg M.S., Takeichi M. (1994). Experimental specification of cell sorting, tissue spreading, and specific spatial patterning by quantitative differences in cadherin expression. Proc. Natl. Acad. Sci. USA.

[100] Reynolds A., Jahoda C. (1992). Cultured dermal papilla cells induce follicle formation and hair growth by transdifferentiation of an adult epidermis. Development.

[101] Inoue K., Kato H., Sato T., Osada A., Aoi N., Suga H., Eto H., Gonda K., Yoshimura K. (2009). Evaluation of Animal Models for the Hair-Inducing Capacity of Cultured Human Dermal Papilla Cells. Cells Tissues Organs.

[102] Osada A., Iwabuchi T., Kishimoto J., Hamazaki T.S., Okochi H. (2007). Long-Term Culture of Mouse Vibrissal Dermal Papilla Cells and *De Novo* Hair Follicle Induction. Tissue Eng..

[103] Kishimoto J., Burgeson R.E., Morgan B.A. (2000). Wnt signaling maintains the hair-inducing activity of the dermal papilla. Genes Dev..

[104] Rendl M., Polak L., Fuchs E. (2008). BMP signaling in dermal papilla cells is required for their hair follicle-inductive properties. Genes Dev..

[105] Toyoshima K.-E., Asakawa K., Ishibashi N., Toki H., Ogawa M., Hasegawa T., Irié T., Tachikawa T., Sato A., Takeda A. (2012). Fully functional hair follicle regeneration through the rearrangement of stem cells and their niches. Nat. Commun..

[106] Kageyama T., Yan L., Shimizu A., Maruo S., Fukuda J. (2019). Preparation of hair beads and hair follicle germs for regenerative medicine. Biomaterials.

[107] Pagani A., Aitzetmüller M.M., Brett E.A., König V., Wenny R., Thor D., Radtke C., Huemer G.M., Machens H.G., Duscher D. (2018). Skin rejuvenation through HIF-1α modulation. Plast. Reconstr. Surg..

[108] Rathman-Josserand M., Genty G., Lecardonnel J., Chabane S., Cousson A., Michelet J.F., Bernard B.A. (2013). Human Hair Follicle Stem/Progenitor Cells Express Hypoxia Markers. J. Investig. Dermatol..

[109] Rezvani H.R., Ali N., Nissen L.J., Harfouche G., De Verneuil H., Taïeb A., Mazurier F. (2011). HIF-1α in epidermis: Oxygen sensing, cutaneous angiogenesis, cancer, and non-cancer disorders. J. Investig. Dermatol..

[110] Yum S., Jeong S., Kim D., Lee S., Kim W., Yoo J.-W., Kim J.A., Kwon O.S., Kim D.D., Min D.S. (2017). Minoxidil induction of VEGF is mediated by inhibition of HIF-prolyl hydrox-ylase. Int. J. Mol. Sci..

[111] Duscher D., Januszyk M., Maan Z.N., Whittam A.J., Hu M.S., Walmsley G.G., Dong Y., Khong S.M., Longaker M.T., Gurtner G.T. (2017). Comparison of the hydroxylase inhibitor DMOG and the iron chelator deferoxamine in diabetic and aged wound healing. Plast. Reconstr. Surg..

[112] Hong W.X., Hu M.S., Esquivel M., Liang G.Y., Rennert R.C., McArdle A., Paik K.J., Duscher D., Gurtner G.C., Lorenz H.P. (2014). The Role of Hypoxia-Inducible Factor in Wound Healing. Adv. Wound Care.

[113] Duscher D., Neofytou E., Wong V.W., Maan Z.N., Rennert R.C., Inayathullah M., Januszyk M., Rodrigues M., Malkovskiy A.V., Whitmore A.J. (2015). Transdermal deferoxamine prevents pressure-induced diabetic ulcers. Proc. Natl. Acad. Sci. USA.

[114] Lim C.H., Sun Q., Ratti K., Lee S.-H., Zheng Y., Takeo M., Lee W., Rabbani P., Plikus M.V., Cain J.E. (2018). Hedgehog stimulates hair follicle neogenesis by creating inductive dermis during murine skin wound healing. Nat. Commun..

[115] Luanpitpong S., Nimmannit U., Pongrakhananon V., Chanvorachote P. (2011). Emblica (Phyllanthus emblica Linn.) fruit extract promotes proliferation in dermal papilla cell of human hair follicle. Res. J. Med. Plant.

[116] Shah C.S., Qudry J.S., Shah Prakashan B.S. (1996). A Text Book of Pharmacognosy.

[117] Jammanesh A., Arbabi Bidgoli S., Ghaffari S., Avadi M.R. (2021). Formulation, characterization and toxicity assessment of *Ginkgo biloba* extract solid lipid nanoparticle in female mice. Nanomed. Res. J..

[118] Saansoomchai P., Limmongkon A., Surangkul D., Chewonarin T., Srikummool M. (2018). Enhanced VEGF expression in hair follicle dermal papilla cells by *Centella asiatica* Linn. Chiang Mai Univ. J. Nat. Sci..

[119] Hay I., Jamieson M., Ormerod A. (1998). The use of aromatherapy as a successful treatment for alopecia areata. J. Eur. Acad. Dermatol. Venereol..

[120] Dhanukar S.A., Thahe U.M. (1989). Therapeutic Approaches. Ayurveda Revisited.

[121] Shimizu K., Kondo R., Sakai K., Shoyama Y., Sato H., Ueno T. (2000). Steroid 5α-Reductase Inhibitory Activity and Hair Regrowth Effects of an Extract from *Boehmeria nipononivea*. Biosci. Biotechnol. Biochem..

[122] Husain A., Virman O.P., Popli S.P., Misra L.N., Gupta M.M., Srivastava G.N., Abraham Z., Singh A.K. (1992). Dictionary of Medicinal Plants.

[123] Nandeesh R., Kumar B.S.A., Lakshman K., Khan S., Swamy V.B.N., Bharathi T., Ganapathy S. (2009). Evaluation of Hair Growth Activity of Buxus wallichiana Baill Extract in Rats. Iran. J. Basic Med. Sci..

[124] Mukerji B.K. (1953). Indian Pharmaceutical Codex.

[125] Dry F.W. (1926). The coat of the mouse (*Mus musculus*). J. Genet..

[126] Shen L., Cui Y. (1998). Effects of the leaf of *Ginkgo biloba* L. extract on blood rheology in animals. Zhongguo Zhong Yao Za Zhi Zhongguo Zhongyao Zazhi China J. Chin. Mater. Medica.

[127] Butler H., Poucher W.A. (1993). Perfumes Cosmetics and Soaps.

[128] Liu W., Xu S., Che C. (2000). Anti-proliferative effect of ginseng saponins on human prostate cancer cell line. Life Sci..

[129] Kobayashi N., Suzuki R., Koide C., Suzuki T., Matsuda H., Kubo M. (1993). Effect of Leaves of *Ginkgo biloba* on Hair Regrowth in C3H Strain Mice. YAKUGAKU ZASSHI J. Pharm. Soc. Jpn..

[130] Mochizuki M., Yoo Y.C., Matsuzawa K., Sato K., Saiki I., Tonooka S., Samukawa K., Azuma I. (1995). Inhibitory effect of tumor metastasis in mice by saponins, ginsenoside-Rb2, 20(R)- and 20(S)-ginsenoside-Rg3, of red ginseng. Biol. Pharm. Bull..

[131] Yadav S.K., Gupta S.K., Prabha S. (2011). Hair growth activity of *Nardostachys jatamansi* and *Cyperus rotundus* rhizomes extract on chemotherapy induced alopecia. Int. J. Drug Dis. Herbal. Res..

[132] Pérez-Ornelas V., Cabeza M., Bratoeff E., Heuze I., Sánchez M., Ramírez E., Naranjo-Rodríguez E. (2005). New 5α-reductase inhibitors: In vitro and in vivo effects. Steroids.

[133] Barbareschi M. (2018). The use of minoxidil in the treatment of male and female androgenetic alopecia: A story of more than 30 years. G. Ital. Dermatol. Venereol..

[134] Mallard C., Louis F., At E. (2015). Cream Gels Comprising at Least One Retnoid and Benzoyl Peroxde. U.S. Patent.

[135] Burry J.S., Evans R.L., Andrew G. (2016). Turner Composition Comprising Azole Fungicide and Water Soluble Metal Salt. EP Patent.

[136] Tanaka F. (2012). Acidic Composition for External Use and Agent for Accelerating Infiltration of Cosmetic Preparation, Hair-Growing Agent, and Preparation for External Use Each Containing the Composition into Skin or the Like. US Patent.

[137] Bosco M., Stucchi L., Fabbian M., Picotti F. (2012). Use of Glycosaminoglycan Lipoate Esters in the Trichology Field. WO Patent.

[138] Gleich P. (2012). Use of a Protease-Containing Hair Growth Reducing Agent. DE Patent.

[139] Renshun G. (2013). Composition for Preventing Hair Loss and Stimulating Hair Growth. CN Patent.

[140] Marinkovich M.P., Gao J., Xu X., Rajadas J. (2013). Methods for Modulating Hair Growth Using Truncated Laminin-511. WO Patent.

[141] Duranton A., Breton L. (2013). Use of Taurine for the Treatment of Alopecia. CA Patent.

[142] Giuliani G., Paus R., Ramot Y., Baroni S., Viti F., Bellinvia S. (2014). Methods of Treating Hair Related Conditions. WO Patent.

[143] Santhanam U., Hong Q., Yim S. (2014). Dickkopf-1 Expression Modulating Compositions and Uses Thereof. US Patent.

[144] Sizhe L. (2014). Chinese Herbal Medicinal Shampoo and Preparation Method for Same. CN Patent.

[145] Kawano M. (2013). Hair Growth Agent/Hair Tonic. EP Patent.

[146] Moser P., Danoux L., Pauly G. (2013). Cosmetic and Pharmaceutical Uses of an Extract of a Plant Belonging to the Genus Buchholzia. US Patent.

[147] Shimazaki A., Shin Y., Yasushi M. (2013). Composition for Hair Growth. WO Patent.

[148] Duran G.A. (2013). Formulation and Method for Treating Hair Loss. WO Patent.

[149] Huchel U., Kropf U., Welss T., Giesen M., Bock A. (2013). Advanced Glycation end Products as Active Ingredients. EP Patent.

[150] Shihong M., Shan K.X., Weiming C.Z. (2013). Formula and Preparation Method of Natural Plant Anti-Hair-Loss and Anti-Dandruff Shampoo. CN Patent.

[151] Ueno R., Habe T., Sekida T. (2014). Composition and Method for Promoting Hair Growth. US Patent.

[152] Bertrand M., Henriat P. (2014). Topical Composition. US Patent.

[153] Chung Y.J., Kim M.U. (2014). Wnt Family-Derived Peptide and Uses Thereof. EP Patent.

[154] Bruning E., Euen T., Gunn G.K., Liebel F., Tucker-Samaras S., VanWyck D., Santora D. (2014). Methods and Compositions for Enhancing Hair Quality Using Blackberry Extract. US Patent.

[155] Kobayashi T., Shizuka U. (2015). Moisturizing Agent. WO Patent.

[156] Price V.H., Menefee E., Strauss P.C. (1999). Changes in hair weight and hair count in men with androgenetic alopecia, after application of 5% and 2% topical minoxidil, placebo, or no treatment. J. Am. Acad. Dermatol..

[157] Matsuda H., Yamazaki M., Naruto S., Asanuma Y., Kubo M. (2002). Antiandrogenic and hair growth promoting activities of Lygodii Spora (spore of *Lygodium japonicum*) I. Active constituents inhibiting testosterone 5 aplhareductase. Biol. Pharm. Bull..

[158] Ali M., Singh V. Phytoconstituents and hair stimulant formulation from *Nordostachys jatamansi*. Proceedings of the International Congress on Traditional Asian Medicine.

[159] Saraf S., Pathak A.K., Dixit V.K. (1991). Hair growth promoting activity of Tridax procumbens. Fitoter.

[160] Sharquie K.E., Al-Obaidi H.K. (2002). Onion Juice (*Allium cepa* L.), A New Topical Treatment for Alopecia Areata. J. Dermatol..

[161] Muradoglu F., Oguz H.I., Yildiz K., Yilmaz H. (2010). Some chemical composition of walnut (*Juglans regia* L.) selections from Eastern Turkey. Afr. J. Agric. Res..

[162] Harada N., Okajima K., Arai M., Kurihara H., Nakagata N. (2007). Administration of capsaicin and isoflavone promotes hair growth by increasing insulin-like growth factor-I production in mice and in humans with alopecia. Growth Horm. IGF Res..

[163] Takahashi T., Kamiya T., Yokoo Y., Hasegawa A. (1999). Procyanidin Oligomers Selectively and Intensively Promote Proliferation of Mouse Hair Epithelial Cells In Vitro and Activate Hair Follicle Growth In Vivo11The authors disclosed conflict of interest. J. Investig. Dermatol..

[164] Takahashi T., Kamimura A., Kagoura M., Toyoda M., Morohashi M. (2005). Investigation of the topical application of procyanidin oligomers from apples to identify their potential use as a hair-growing agent. J. Cosmet. Dermatol..

[165] Hsu S. (2005). Green tea and the skin. J. Am. Acad. Dermatol..

[166] Philpott M.P., Kealey T. (2000). Cyclical Changes in Rat Vibrissa Follicles Maintained In Vitro. J. Investig. Dermatol..

[167] Wikramanayake T.C., Rodriguez R., Choudhary S., Mauro L.M., Nouri K., Schachner L.A., Jimenez J.J. (2012). Effects of the Lexington LaserComb on hair regrowth in the C3H/HeJ mouse model of alopecia areata. Lasers Med. Sci..

[168] Hoffmann R., Happle R. (2000). Current understanding of androgenetic alopecia. Part II: Clinical aspects and treatment. Eur. J. Dermatol..

[169] Zarei M., Wikramanayake T.C., Falto-Aizpurua L., Schachner L.A., Jimenez J.J. (2016). Low level laser therapy and hair regrowth: An evidence-based review. Lasers Med. Sci..

[170] Wikramanayake T.C., Villasante A.C., Mauro L.M., Nouri K., Schachner L.A., Perez C.I., Jimenez J.J. (2012). Low-level laser treatment accelerated hair regrowth in a rat model of chemotherapy-induced alopecia (CIA). Lasers Med. Sci..

[171] Gilhar A., Shalaginov R., Assy B., Serafimovich S., Kalish R.S. (1999). Alopecia areata is a T-lymphocyte mediated autoimmune disease: Lesional human T-lymphocytes transfer alopecia areata to human skin grafts on SCID mice. Journal of Investigative Dermatology Symposium Proceedings.

[172] Orasan M.S., Roman I.I., Coneac A., Muresan A., Orasan R.I. (2016). Hair loss and regeneration performed on animal models. Clujul Medical.

[173] Van Neste D., de Brouwer B. (2000). Human hair follicle grafts in nude mice: An important in vivo model for investigating the control of hair growthp. Hair and Its Disorders: Biology, Pathology and Management.

[174] Paus R., Stenn K.S., Link R.E. (1989). The induction of anagen hair growth in telogen mouse skin by cyclosporine A administration. Labor. Investig. J. Tech. Methods Pathol..

[175] Müller-Röver S., Foitzik K., Paus R., Handjiski B., van der Veen C., Eichmüller S., McKay I.A., Stenn K.S. (2001). A Comprehensive Guide for the Accurate Classification of Murine Hair Follicles in Distinct Hair Cycle Stages. J. Investig. Dermatol..

[176] Kloepper J.E., Sugawara K., Al-Nuaimi Y., Gáspár E., van Beek N., Paus R. (2010). Methods in hair research: How to objectively distinguish between anagen and catagen in human hair follicle organ culture. Exp. Dermatol..

[177] Gnann L.A., Castro R.F., Azzalis L.A., Feder D., Perazzo F.F., Pereira E.C., Rosa P.C.P., Junqueira V.B.C., Rocha K.C., Machado C.D.A. (2013). Hematological and hepatic effects of vascular epidermal growth factor (VEGF) used to stimulate hair growth in an animal model. BMC Dermatol..

[178] Singh S., Shukla V.K. (2021). Current regulations for Herbal Medicines in India. Int. J. Drug Regul. Aff..

[179] Santos A.C., Ferreira C., Veiga F., Ribeiro A.J., Panchal A., Lvov Y., Agarwal A. (2018). Halloysite clay nanotubes for life sciences applications: From drug encapsulation to bioscaffold. Adv. Colloid Interface Sci..

[180] Mavridi-Printezi A., Guernelli M., Menichetti A., Montalti M. (2020). Bio-Applications of Multifunctional Melanin Nanoparticles: From Nanomedicine to Nanocosmetics. Nanomaterials.

[181] Liu M., Jia Z., Jia D., Zhou C. (2014). Recent advance in research on halloysite nanotubes-polymer nanocomposite. Prog. Polym. Sci..

[182] Abdullayev E., Joshi A., Wei W., Zhao Y., Lvov Y. (2012). Enlargement of Halloysite Clay Nanotube Lumen by Selective Etching of Aluminum Oxide. ACS Nano.

[183] Santos A.C., Panchal A., Rahman N., Pereira-Silva M., Pereira I., Veiga F., Lvov Y. (2019). Evolution of Hair Treatment and Care: Prospects of Nanotube-Based Formulations. Nanomaterials.

[184] Asenov A., Oliveira F.A., Speare R., Liesenfeld O., Hengge U.R., Heukelbach J. (2010). Efficacy of chemical and botanical over-the-counter pediculicides available in Brazil, and off-label treatments, against head lice ex vivo. Int. J. Dermatol..

[185] Downs A.M., Stafford K.A., Hunt L.P., Ravenscroft J.C., Coles G.C. (2002). Widespread insecticide resistance in head lice to the over-the-counter pediculocides in England, and the emergence of carbaryl resistance: Therapeutics. Br. J. Dermatol..

[186] Tian L., Li X., Ji H., Yu Q., Yang M., Guo L., Huang L., Gao W. (2022). Melanin-like nanoparticles: Advances in surface modification and tumour photothermal therapy. J. Nanobiotechnology.

[187] Sentamilselvi G., Janaki C., Murugusundram S. (2009). Trichomycoses. Int. J. Trichol..

[188] Chanprapaph K., Udompanich S., Visessiri Y., Ngamjanyaporn P., Suchonwanit P. (2019). Nonscarring alopecia in systemic lupus erythematosus: A cross-sectional study with trichoscopic, histopathologic, and immunopathologic analyses. J. Am. Acad. Dermatol..

[189] Goyal R., Macri L.K., Kaplan H.M., Kohn J. (2016). Nanoparticles and nanofibers for topical drug delivery. J. Control. Release.

[190] Rosen J., Landriscina A., Friedman A.J. (2015). Nanotechnology-Based Cosmetics for Hair Care. Cosmetics.

[191] Chen P., Miao Y., Zhang F., Huang J., Chen Y., Fan Z., Yang L., Wang J., Hu Z. (2020). Nanoscale microenvironment engineering based on layer-by-layer self-assembly to regulate hair follicle stem cell fate for regenerative medicine. Theranostics.

[192] Enyiğit T., Sonvico F., Rossi A., Tekmen I., Santi P., Colombo P., Nicoli S., Özer Ö. (2016). In vivo assessment of clobetasol propionate-loaded lecithin-chitosan nanoparticles for skin delivery. Int. J. Mol. Sci..

[193] Matos B.N., Reis T., Gratieri T., Gelfuso G.M. (2015). Chitosan nanoparticles for targeting and sustaining minoxidil sulphate delivery to hair follicles. Int. J. Biol. Macromol..

[194] Lee S., Zürcher S., Dorcier A., Luengo G.S., Spencer N.D. (2009). Adsorption and Lubricating Properties of Poly(l-lysine)-*graft*-poly(ethylene glycol) on Human-Hair Surfaces. ACS Appl. Mater. Interfaces.

[195] Leal Cardoso J.H., Noronha Coelho de Souza A., Militão de Souza F., Sa Preire S., Pinçon C. (2020). Treatment of Head Louse Infestation with a Novel Mixture Made of Semi-Crystalline Polymers and Plant Extracts: Blind, Randomized, Controlled, Superiority Trial. Cosmetics.

[196] Wang W., Chen L., Huang X., Shao A. (2016). Preparation and Characterization of Minoxidil Loaded Nanostructured Lipid Carriers. AAPS PharmSciTech.

[197] Kumar R., Singh B., Bakshi G., Katare O.P. (2007). Development of Liposomal Systems of Finasteride for Topical Applications: Design, Characterization, and In Vitro Evaluation. Pharm. Dev. Technol..

[198] Haveli S.D., Walter P., Patriarche G., Ayache J., Castaing J., Van Elslande E., Tsoucaris G., Wang P.A., Kagan H.B. (2012). Hair fiber as a nano-reactor in controlled synthesis of fluorescent gold nanoparticles. Nano Lett..

[199] Marimuthu S., Rahuman A.A., Santhoshkumar T., Jayaseelan C., Kirthi A.V., Bagavan A., Kamaraj C., Elango G., Zahir A.A., Rajakumar G. (2012). Lousicidal activity of synthesized silver nanoparticles using *Lawsonia inermis* leaf aqueous extract against *Pediculus humanus capitis* and *Bovicola ovis*. Parasitol. Res..

[200] Al Mahrooqi J.H., Khutoryanskiy V.V., Williams A.C. (2021). Thiolated and PEGylated silica nanoparticle delivery to hair follicles. Int. J. Pharm..

[201] Zhang Y., Chang M., Bao F., Xing M., Wang E., Xu Q., Huan Z., Guo F., Chang J. (2019). Multifunctional Zn doped hollow mesoporous silica/polycaprolactoneelectrospun membranes with enhanced hair follicle regeneration and antibacterial activity for wound healing. Nanoscale.

[202] Cavallaro G., Milioto S., Konnova S., Fakhrullina G., Akhatova F., Lazzara G., Fakhrullin R., Lvov Y. (2020). Halloysite/Keratin Nanocomposite for Human Hair Photoprotection Coating. ACS Appl. Mater. Interfaces.

[203] Leerunyakul K., Suchonwanit P. (2020). Asian hair: A review of structures, properties, and distinctive disorders. Clin. Cosmet. Investig. Dermatol..

[204] Guryanov I., Naumenko E., Fakhrullin R. (2022). Hair surface engineering: Combining nanoarchitectonics with hair topical and beauty formulations. Appl. Surf. Sci. Adv..

[205] Pucek A., Tokarek B., Waglewska E., Bazylińska U. (2020). Recent Advances in the Structural Design of Photosensitive Agent Formulations Using “Soft” Colloidal Nanocarriers. Pharmaceutics.

[206] Nazir H., Wang L., Lian G., Zhu S., Zhang Y., Liu Y., Ma G. (2012). Multilayered silicone oil droplets of narrow size distribution: Preparation and improved deposition on hair B. Biointerfaces.

[207] Yuan W., Hu Z., Liao M., Cai Y., Meng L., Liu Z., Chen Y., Liu Y., Lu N. (2012). A novel preparation method for silicone oil nanoemulsions and its application for coating hair with silicone. Int. J. Nanomed..

[208] Sonneville-Aubrun O., Simonnet J.T., L’alloret F. (2004). Nanoemulsions: A new vehicle for skincare products. Adv. Coll. Interface Sci..

[209] Gavazzoni Dias M.F. (2015). Hair cosmetics: An overview. Int. J. Trichology.

[210] Song C., Liu S. (2005). A new healthy sunscreen system for human: Solid lipid nannoparticles as carrier for 3,4,5-trimethoxybenzoylchitin and the improvement by adding Vitamin E. Int. J. Biol. Macromol..

[211] Nogueira A.C.S., Joekes I. (2004). Hair color changes and protein damage caused by ultraviolet radiation. J. Photochem. Photobiol. B Biol..

[212] Panchal A., Fakhrullina G., Fakhrullin R., Lvov Y. (2018). Self-assembly of clay nanotubes on hair surface for medical and cosmetic formulations. Nanoscale.

[213] Cavallaro G., Lazzara G., Milioto S., Parisi F., Evtugyn V.G., Rozhina E., Fakhrullin R.F. (2018). Nanohydrogel Formation within the Halloysite Lumen for Triggered and Sustained Release. ACS Appl. Mater. Interfaces.

[214] Tarasova E., Naumenko E., Rozhina E., Akhatova F., Fakhrullin R. (2019). Cytocompatibility and uptake of polycations-modified halloysite clay nanotubes. Appl. Clay Sci..

[215] Guryanov I., Naumenko E., Akhatova F., Lazzara G., Cavallaro G., Nigamatzyanova L., Fakhrullin R. (2020). Selective cytotoxic activity of prodigiosin@ halloysitenanoformulation. Front. Bioeng. Biotechnol..

[216] Naumenko E.A., Guryanov I.D., Yendluri R., Lvov Y.M., Fakhrullin R.F. (2016). Clay nanotube–biopolymer composite scaffolds for tissue engineering. Nanoscale.

[217] Rahman N., Scott F.H., Lvov Y., Stavitskaya A., Akhatova F., Konnova S., Fakhrullina G., Fakhrullin R. (2021). Clay Nanotube Immobilization on Animal Hair for Sustained Anti-Lice Protection. Pharmaceutics.

[218] Nanda A., Nanda S., Nguyen T.A., Slimani Y., Rajendran S. (2020). Nanocosmetics: Fundamentals, Applications and Toxicity.

[219] Thun M.J., Altekruse S.F., Namboodiri M.M., Calle E.E., Myers D.G., Heath C.W. (1994). Hair dye use and risk of fatal cancers in US women. J. Natl. Cancer Inst..

[220] Baki G., Alexander K.S. (2015). Introduction to Cosmetic Formulation and Technology.

[221] Lee H.Y., Jeong Y.I., Choi K.C. (2011). Hair dye-incorporated poly-γ-glutamic acid/glycol chitosan nanoparticles based on ion-complex formation. Int. J. Nanomed..

[222] Im K.M., Kim T.-W., Jeon J.-R. (2017). Metal-Chelation-Assisted Deposition of Polydopamine on Human Hair: A Ready-to-Use Eumelanin-Based Hair Dyeing Methodology. ACS Biomater. Sci. Eng..

[223] Gao Z.F., Wang X.Y., Gao J.B., Xia F. (2019). Rapid preparation of polydopamine coating as a multifunctional hair dye. RSC Adv..

[224] Trelles M.A., Almudever P., Alcolea J.M., Cortijo J., Serrano G., Expósito I., Royo J., Leclère F.M. (2016). Cuttlefish Ink Melanin Encapsulated in Nanolipid Bubbles and Applied Through a Micro-Needling Procedure Easily Stains White Hair Facilitating Photoepilation. J. Drugs Dermatol..

[225] Gourlaouen L., Lee K. (2004). Composition and Method of Dyeing Keratin Fibers Comprising Luminescent Semiconductive Nanoparticles. U.S. Patent.

[226] Luo C., Zhou L., Chiou K., Huang J. (2018). Multifunctional Graphene Hair Dye. Chem.

[227] Gandhi P.R., Jayaseelan C., Mary R.R., Mathivanan D., Suseem S.R. (2017). Acaricidal, pediculicidal and larvicidal activity of synthesized ZnO nanoparticles using Momordicacharantia leaf extract against blood feeding parasites. Exp. Parasitol..

[228] Lebwohl M., Clark L., Levitt J. (2007). Therapy for Head Lice Based on Life Cycle, Resistance, and Safety Considerations. Pediatrics.

[229] Morganti P., Palombo M., Cardillo A., Del Ciotto P., Morganti G., Gazzaniga G. (2012). Anti-dandruff and anti-oily efficacy of hair formulations with a repairing and restructuring activity. The positive influence of the Zn-chitin nanofibrils complexes. J. Appl. Cosmetol..

[230] Lamore S.D., Cabello C.M., Wondrak G.T. (2009). The topical antimicrobial zinc pyrithione is a heat shock response inducer that causes DNA damage and PARP-dependent energy crisis in human skin cells. Cell Stress Chaperon.

[231] Gao W., Thamphiwatana S., Angsantikul P., Zhang L. (2014). Nanoparticle approaches against bacterial infections. Wiley Interdiscip. Rev. Nanomed. Nanobiotechnol..

[232] Zakharova O.V., Godymchuk A.Y., Gusev A.A., Gulchenko S.I., Vasyukova I.A., Kuznetsov D.V. (2015). Considerable Variation of Antibacterial Activity of Cu Nanoparticles Suspensions Depending on the Storage Time, Dispersive Medium, and Particle Sizes. BioMed Res. Int..

[233] Shrivastava S., Bera T., Roy A., Singh G., Ramachandrarao P., Dash D. (2007). Characterization of enhanced antibacterial effects of novel silver nanoparticles. Nanotechnology.

[234] Wang L., Hu C., Shao L. (2017). The antimicrobial activity of nanoparticles: Present situation and prospects for the future. Int. J. Nanomed..

[235] Rilda Y., Damara D., Putri Y.E., Refinel R., Agustien A., Pardi H. (2020). Pseudomonas aeruginosa antibacterial textile cotton fiber construction based on ZnO–TiO2 nanorods template. Heliyon.

[236] Kaul S., Gulati N., Verma D., Mukherjee S., Nagaich U. (2018). Role of Nanotechnology in Cosmeceuticals: A Review of Recent Advances. J. Pharm..

[237] Vickers N.J. (2017). Animal communication: When I’m calling you, will you answer too?. Curr. Biol..

[238] Singh I., Dhawan G., Gupta S., Kumar P. (2021). Recent Advances in a Polydopamine-Mediated Antimicrobial Adhesion System. Front. Microbiol..

[239] Liu C.-Y., Huang C.-J. (2016). Functionalization of Polydopamine via the Aza-Michael Reaction for Antimicrobial Interfaces. Langmuir.

[240] Liu H., Qu X., Tan H., Song J., Lei M., Kim E., Payne G.F., Liu C. (2019). Role of polydopamine’s redox-activity on its pro-oxidant, radical-scavenging, and antimicrobial activities. Actabiomaterialia.

[241] Smith A., Perelman M., Hinchcliffe M. (2014). Chitosan: A promising safe and immune-enhancing adjuvant for intranasal vaccines. Hum. Vaccines Immunother..

[242] Azuma K., Koizumi R., Izawa H., Morimoto M., Saimoto H., Osaki T., Ito N., Yamashita M., Tsuka T., Imagawa T. (2018). Hair growth-promoting activities of chitosan and surface-deacetylated chitin nanofibers. Int. J. Biol. Macromol..

[243] Zhao D., Yu S., Sun B., Gao S., Guo S., Zhao K. (2018). Biomedical Applications of Chitosan and Its Derivative Nanoparticles. Polymers.

[244] Kravanja G., Primožič M., Knez Ž., Leitgeb M. (2019). Chitosan-Based (Nano)Materials for Novel Biomedical Applications. Molecules.

[245] Pereira M.N., Ushirobira C.Y., Cunha-Filho M.S., Gelfuso G.M., Gratieri T. (2018). Nanotechnology advances for hair loss. Ther. Deliv..

[246] Gupta A., Aggarwal G., Singla S., Arora R. (2012). Transfersomes: A Novel Vesicular Carrier for Enhanced Transdermal Delivery of Sertraline: Development, Characterization, and Performance Evaluation. Sci. Pharm..

[247] Pelikh O., Eckert R.W., Pinnapireddy S.R., Keck C.M. (2020). Hair follicle targeting with curcumin nanocrystals: Influence of the formulation properties on the penetration efficacy. J. Control. Release.

[248] Vidlářová L., Romero G.B., Hanuš J., Štěpánek F., Müller R.H. (2016). Nanocrystals for dermal penetration enhancement—Effect of concentration and underlying mechanisms using curcumin as model. Eur. J. Pharm. Biopharm..

